# Signal-Enhanced Electrochemical Determination of Quercetin with Poly(chromotrope fb)-Modified Pencil Graphite Electrode in Vegetables and Fruits

**DOI:** 10.1021/acsomega.3c00599

**Published:** 2023-03-24

**Authors:** Lokman Liv, Erman Karakuş

**Affiliations:** † Electrochemistry Laboratory, Chemistry Group, The Scientific and Technological Research Council of Turkey, National Metrology Institute, (TUBITAK UME), 41470 Gebze, Kocaeli, Turkey; ‡ Organic Chemistry Laboratory, Chemistry Group, The Scientific and Technological Research Council of Turkey, National Metrology Institute, (TUBITAK UME), 41470 Gebze, Kocaeli, Turkey

## Abstract

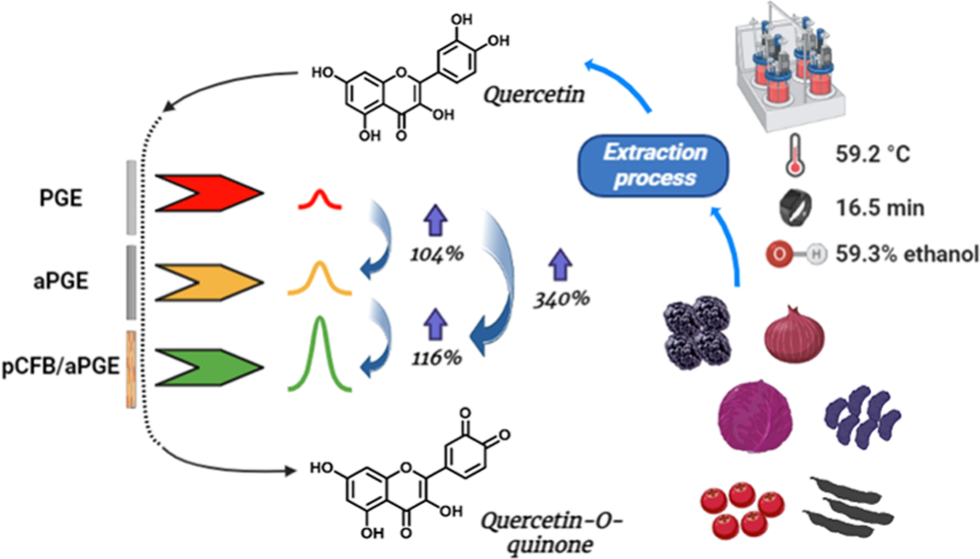

A novel signal-enhanced electrochemical sensing strategy was constructed for quercetin determination with a peculiarly developed poly(chromotrope fb)-modified activated pencil graphite electrode in vegetables and fruits. The oxidation signal of quercetin at 118 mV in an alcoholic solution served as the analytical response. The produced platform, characterized by cyclic voltammetry, electrochemical impedance spectroscopy, scanning electron microscopy, energy-dispersive X-ray spectroscopy, and X-ray photoelectron spectroscopy, could detect 1.9 nM of quercetin in the range of 0.01–1.2 μM. The extracted quercetin contents of red onion, red cabbage, cranberry, black mulberry, black raisin, and carob were determined by both the developed method and UV–visible spectroscopy. The results were statistically evaluated at the 95% confidence level, and no significant difference between the results was found.

## 1 Introduction

Quercetin (Qn, 3,3′,4′,5,7-pentahydroxyflavone), also named as vitamin P, is a significant carbohydrate-free bioflavonoid that is generally found in plants, vegetables, and fruits.^1^ Qn is a bioactive flavonoid with many therapeutic properties in diabetes, cardiovascular and arthritic diseases, autophagy, and Alzheimer’s disease.^2^ In addition, it has antioxidant, antimicrobial, anti-inflammatory, antiallergic, antiobesity, antitumor, antihypertensive, antihypercholesterolemic, antiatherosclerotic, immunomodulatory, neuroprotective, and vasodilator effects.^3^

Qn has shown many biological benefits including the propitiation of morning stiffness and pain in rheumatoid arthritis patients, inhibition of cytokine production, reducing lipoxygenase and cyclooxygenase expression, providing the stability of mast cells,^4^ restraint of the cytopathic effects of rhinovirus, echovirus, coxsackievirus, and poliovirus, decreasing the formation of RNA and DNA viruses involving respiratory syncytial virus, polio type 1, parainfluenza type 3, and herpes simplex virus-1,^5^ protecting brain cells against oxidative stress and excitotoxicity and thus preventing Alzheimer’s disease,^6^ preventing cancer- with apoptosis-inducing effects, promoting the insulin-sensitizing effect to hinder diabetes and inhibiting platelet aggregation, and helping to impede cardiovascular diseases by improving the health of the endothelium.^7^

It is recommended to take 500–1000 mg/day, which should be included in the daily diet,^8^ whereas the overdose may cause headache, inflammation, and damage to the kidney and DNA structure.^8,9^ Thus, considering both the advantages and disadvantages, monitoring of the Qn level in foodstuffs is very significant. The methods based on spectrophotometry^10,11^ and chromatography^12,13^ are widely used for determining Qn. Although these methods yield in terms of sensitivity and selectivity, they employ labor-intensive pretreatment steps, difficult separation procedures, and sophisticated and expensive instruments.^14,15^ In recent years, electrochemical sensors have come to the forefront for determining some electroactive food and biologically essential substances.^16−18^ Due to some distinct features of electrochemical methods such as being simple, quick, portable, cost-effective, sensitive, and selective, they have become one of the most encouraging methods for determining Qn.^19,20^ A wide variety of modified electrodes including single- or multipolymer film-modified electrodes with or without metal nanoparticles,^21−25^ carbon nanotube-based electrodes with^26^ or without polymer films,^27,28^ molecularly imprinted polymer (MIP)-based electrodes,^29,30^ and the other ones^31,32^ have been reported for the determination of Qn, and they are summarized in Table S1. The electrodes composed of a single polymer^21,24^ have high limit of detection (LOD) values (i.e., 0.17^21^ and 20 μM^24^); the other electrodes have complex and time-consuming preparation steps,^22,23,25−32^ and the supporting surfaces in these studies, i.e., glassy carbon electrodes (GCEs), carbon paste electrodes (CPEs), and paraffin-impregnated graphite disk electrode, have advantages such as being prone to electrocatalysis, resistant to surface contamination, and undesired electrode reactions.^33−38^ However, they have disadvantages due to being expensive, nondisposable, and requiring pretreatment. On the other hand, pencil graphite electrodes (PGEs) have some benefits involving cheapness, common technology, good electrochemical reactivity and mechanical endurance, and being disposable, commercially available, renewable, and suitable for modification.^14,15,19,20^ It has been reported that the polymer formed as a result of the uniform arrangement of azo dyes containing a hydroxyl group in the ortho position to the azoic group forms a stable redox-active layer, increases the electrode active surface area, and has an electrocatalytic effect toward the oxidation of small organic molecules with adjacent hydroxyl groups such as dopamine, ascorbic acid, and uric acid.^39,40^ In view of these findings, a novel electrochemical sensing platform based on poly(chromotrope fb)-modified activated pencil graphite electrode (pCFB/aPGE) that overcomes the mentioned limitations was produced for the determination of Qn in vegetables and fruits. Additionally, this is the first instance in which chromotrope fb (CFB) was electropolymerized. It is also the first example of the voltammetric determination of Qn in red cabbage, cranberry, black mulberry, black raisin, and carob.

## 2 Materials and Methods

### 2.1 Chemicals and Equipment

Chromotrope fb (CFB, Alfa Aesar B22328), quercetin (Qn, Sigma-Aldrich Q4951, ≥95%), methanol (Sigma-Aldrich 494437, BioReagent, ≥99.93%), sodium hydroxide (Merck 1.06498, Emsure, ≥99%), potassium dihydrogen phosphate (Merck 1.04873, Emsure, ≥99.5%), potassium chloride (Sigma-Aldrich P3911, ACS Reagent, 99.0–100.5%), d-glucose (Sigma-Aldrich G8270, ≥99.5%), sucrose (Sigma-Aldrich S0389, ≥99.5%), dopamine hydrochloride (TCI A0305, >98.0%), l-cysteine (Sigma-Aldrich C7352, ≥98%), l-ascorbic acid (Sigma-Aldrich A5960, BioXtra, ≥99.0%), glycine (Merck 1.04201, ≥99.7%), citric acid (Merck 8.18707, ≥99.0%), sodium chloride (Merck 1.06406, Suprapur, 99.99%), sodium nitrite (Merck 1.06549, Emsure, ≥99.0%), sodium perchlorate (Sigma-Aldrich 410241, ACS Reagent, ≥98.0%), calcium chloride (Alfa Aesar L13191, 97%), iron(III) chloride (Sigma-Aldrich 8.03945), aluminum chloride (Alfa Aesar A11892, 99%), magnesium chloride (Sigma-Aldrich M8266, ≥98.0%), sodium sulfate (Merck 1.06643, Emprove, 99.0–100.5%), sodium nitrate (Sigma-Aldrich S5506, ReagentPlus, ≥99.0%), sodium thiosulfate (Sigma-Aldrich 217263, ReagentPlus, 99%), and methanol (Merck 1.06035, Supelco, ≥99.97%) were used as analytical reagent grades. The Qn solution was freshly prepared in methanol; the other solutions were prepared in ultrapure water, and all of them were stored in high-density polyethylene falcon tubes. Merck Millipore Milli-Q Integral 10 system was operated to produce ultrapure water.

Voltammetric measurements were carried out with Metrohm Autolab PGSTAT 128N potentiostat/galvanostat with a conventional three-electrode system consisting of a poly(chromotrope fb)-modified activated pencil graphite electrode (pCFB/aPGE, pencil body: ONAS MP 775, supporting surface: Tombow 2H 0.5 mm, surface area: 15.90 mm^2^, immersion depth: 1 cm), a Ag/AgCl electrode (inner solution: 3 M NaCl, BASi MF-2052 RE-5B), and a platinum wire (BASi MW-1032, 7.5 cm) as the working, reference, and counter electrodes, respectively. The other grades of graphite leads (i.e., H, HB, B, 2B, and 3B) were purchased from a stationary in Kocaeli. Electrochemical impedance spectroscopy (EIS) measurements were performed with the FRA32M Impedance Module connected to a Metrohm Autolab PGSTAT 128N potentiostat/galvanostat.

UV–visible measurements were performed with a UV–visible spectrophotometer (Shimadzu UV-1900i). A Mettler Toledo Seven Easy pH meter with an InLab Expert Pro probe was used to prepare buffer solutions. A Hitachi Schottky SU5000 field emission-scanning electron microscope (SEM), an FEI Oxford Instruments Model 7260 energy-dispersive X-ray spectroscopy (EDX), and a Thermo Fisher K-α X-ray photoelectron spectroscopy (XPS) were used to characterize the pCFB/aPGE.

### 2.2 Preparation of pCFB/aPGE

**Figure 1:**
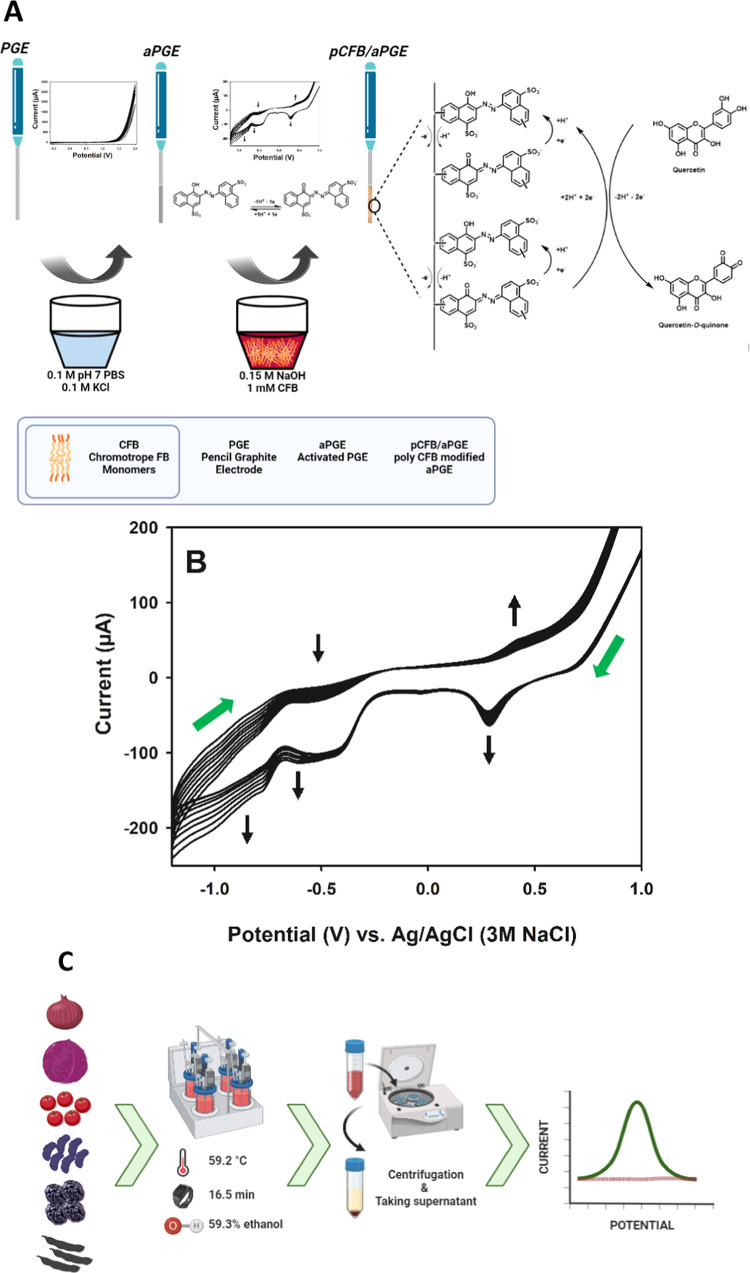
(A) Preparation procedure of pCFB/aPGE and plausible mechanism of electrochemical detection, (B) electropolymerization voltammograms of pCFB/aPGE, and (C) schematic illustration of the sample preparation and measurement procedure.

The preparation procedure of pCFB/aPGE and the plausible mechanism of electrochemical detection and electropolymerization voltammograms of pCFB/aPGE are shown in Figure 1A,B, respectively. First, the bare PGE was activated in 0.1 M of pH 7 phosphate buffer solution (PBS) and 0.1 M KCl via cycling 5 times between −0.6 and 2.0 V with 50 mV/s of scan rate^41^ and denoted as aPGE. Then, CFB (1 mM) was electropolymerized onto the aPGE in 0.15 M of NaOH solution via cycling 20 repetitive scans between −1.2 and 1.6 V with a scan rate of 100 mV/s. In both cases, the total volume of the solution was adjusted to 10 mL. The peak current increase and decrease in the related regions indicate that the electropolymerization has taken place, as shown in Figure 1B. The final platform was stated as pCFB/aPGE and prepared daily.

### 2.3 Voltammetric Measurement Procedure

Differential pulse voltammetry (DPV) was anodically swept between −0.4 and 1 V with step and pulse amplitudes of 5 and 25 mV, respectively, and a scan rate of 25 mV/s. Cyclic voltammetry (CV) was applied in the respective potential intervals (−0.7–1.1 V for surface characterization in 1 mM of K_3_[Fe(CN)_6_], 1 mM of K_4_[Fe(CN)_6_] and 0.1 M of KCl and −1 to 1 V for the appearance of electrocatalytic effect and CV characteristics in the presence of Qn) with a step amplitude of 3 mV and a proper scan rate. The solution consisting of 0.03 M pH 7 PBS with or without the vegetable–fruit extract was adjusted to 10 mL with ultrapure water and degassed with argon for 3 min before the measurement.

### 2.4 UV–Visible Spectroscopy Measurement Procedure

The solutions of Qn (1 mM) and vegetable–fruit samples (50-fold diluted) were prepared in ethanol. For each sample, a 1:1 (v/v) ethanol:water mixture containing vegetable or fruit sample and a known concentration of Qn (2.5, 5.0, 7.5, and 10.0 μM) were prepared. UV–visible measurements were carried out in 10.0 mm path-length quartz cuvettes with 700 μL of volume. The absorption spectra were examined in the range of 300–700 nm with six replicates for each sample.

### 2.5 Sample Preparation Procedure

Qn extraction was performed with minor modifications to reference.^42^ In summary, 200 μM of Qn (for 50 mL) was added to the samples before the extraction process. Then, the extraction of Qn was initiated by mixing 5 g of separately ground red onion, red cabbage, cranberry, black mulberry, black raisin, and carob with 40 mL of 59.3% ethanol and continued in a shaking incubator at 59.2 °C for 16.5 min. After centrifugation at 1600*g* for 30 min, the supernatants of vegetable and fruit samples were separated, and the final volume for each supernatant was adjusted to 50 mL with ultrapure water and tested to analyze the yield of quercetin, as shown in Figure 1C. The supernatants were further diluted 500-fold (20 μL/10 mL, approx. 400 nM of Qn) and analyzed with voltammetry. On the other hand, the supernatants were further diluted 40-fold (25 μL/1 mL, approx. 5 μM of Qn) and analyzed with a UV–visible spectroscopy.

## 3 Results and Discussion

### 3.1 Surface Characterization of pCFB/aPGE

**Figure 2:**
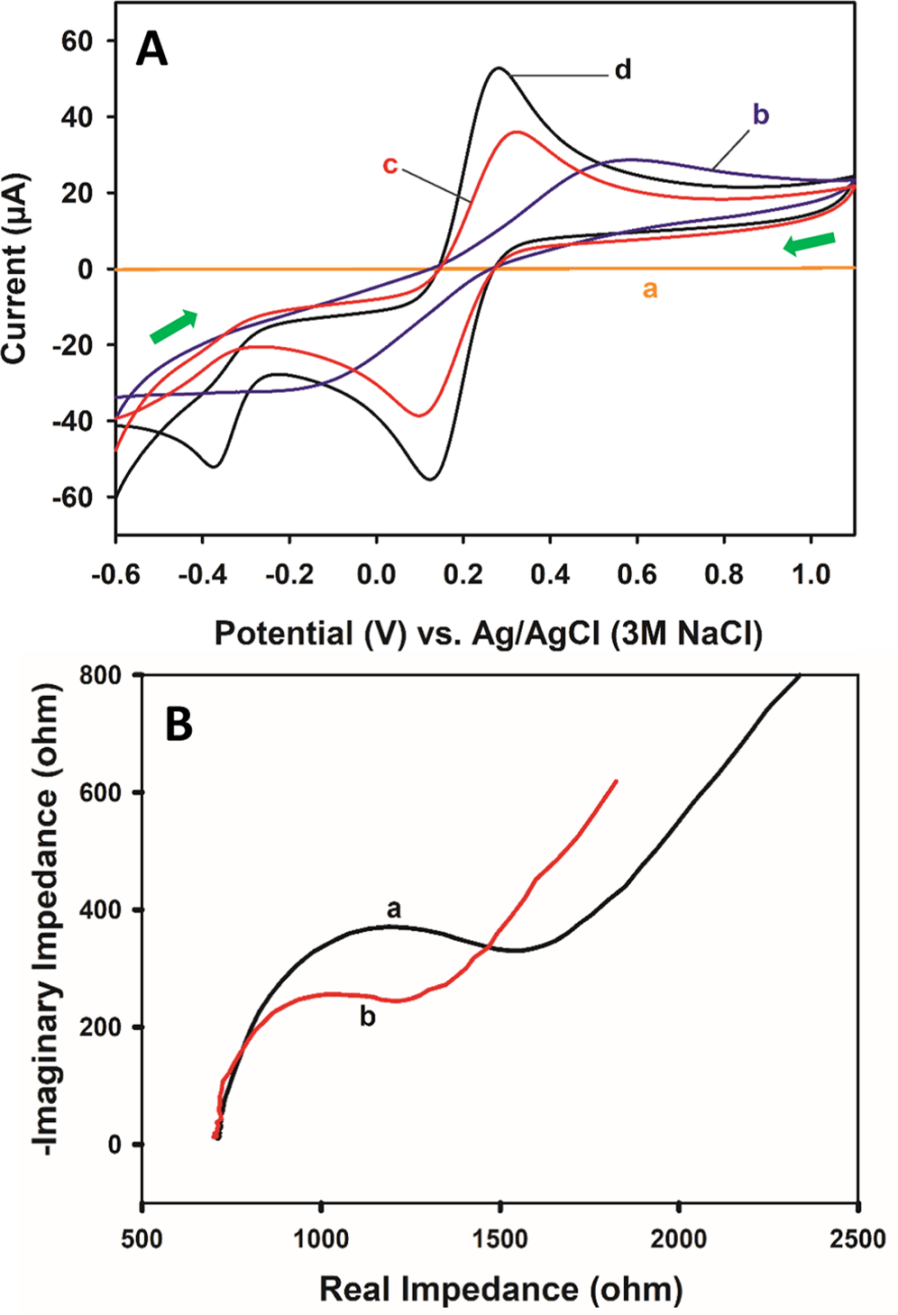
(A) Cyclic voltammograms of (a) bare PGE, (b) pCFB/PGE, (c) aPGE, and (d) pCFB/aPGE with a scan rate of 50 mV/s and (B) EIS spectra of (a) aPGE and (b) pCFB/aPGE within a frequency range of 0.1–100,000 Hz in the presence of 1 mM of K_3_[Fe(CN)_6_], 1 mM of K_4_[Fe(CN)_6_], and 0.1 M of KCl.

The characterization of the bare PGE, pCFB-modified bare PGE (pCFB/PGE; with only CV and EIS), aPGE, and pCFB/aPGE was investigated with CV, EIS, SEM, EDX, and XPS. First, CV measurements were performed in the presence of 1 mM of K_3_[Fe(CN)_6_], 1 mM of K_4_[Fe(CN)_6_], and 0.1 M of KCl, as shown in Figure 2A. No redox peak was obtained with the bare PGE (Figure 2A-a), while an oxidation peak at 0.60 V and a reduction peak at −0.10 V belonging to the [Fe(CN)_6_]^4–^/[Fe(CN)_6_]^3–^ couple were observed with the pCFB/PGE (Figure 2A-b). With the activation of the bare PGE, the reversibility of the system improved and the peak currents increased significantly (Figure 2A-c). With pCFB modified on the aPGE, the reversibility of the system improved by about 35 mV and the peak currents increased by 48% compared to aPGE (Figure 2A-d). The peak at −0.37 V resulted from the interaction between the surface of pCFB/aPGE and the redox couple.

EIS spectra of the aPGE and pCFB/aPGE are shown in Figure 2B, while EIS spectra of the bare PGE and pCFB/PGE are shown in Figures S1 and S2, respectively. EIS spectra with the *R*_s_(*C*_dl_[*R*_ct_W]) circuit yielded *R*_s_ (i.e., solution resistance), *C*_dl_ (i.e., double-layer capacitance), *R*_ct_ (i.e., charge-transfer resistance), and *W* (i.e., Warburg impedance) values, indicating that the pCFB/aPGE had great electron-transfer rate (21.9 μF) and low charge-transfer resistance (459 Ω) compared to the aPGE (8.92 μF and 716 Ω). In addition, real impedance was plotted against the square root of the inverse of the radial frequency (ω^–0.5^), and the slope values of 0.0011 for aPGE (Figure S3A) and 0.0016 for pCFB/aPGE (Figure S3B) obtained from the linear parts of the curves (low-frequency region) demonstrate that the diffusion ability of pCFB/aPGE is better than that of aPGE.^43^ These results confirm that the EIS results are in agreement with the CV results.

In addition to all, the absence of redox peaks or low intensity and poor reversibility in Figure 2A-a,b coincides with the EIS spectra in Figures S1 and S2, for which it is not possible to simulate the relevant circuit.

SEM images for the bare PGE, aPGE, and pCFB/aPGE appear in Figure S4. It is seen in Figure S4A that the bare PGE surface does not show a uniform distribution, while the aPGE has a more uniform and channeled appearance (Figure S4B). After the electropolymerization, it is clearly observed that these channels are filled with pCFB (Figure S4C).

EDX spectra of the bare PGE, aPGE, and pCFB/aPGE are shown in Figure S5. It is observed that PGE contains aluminum (1.2%) due to the aluminum oxide in the structure of pencil graphite in addition to carbon (96.2%) and oxygen (2.6%) (Figure S5A).^44^ With the activation of bare PGE, the amount of oxygen increased approximately 3 times (7.9%) and sodium (0.5%), phosphorus (0.3%), and potassium (0.5%) appeared probably due to the phosphate buffer (KH_2_PO_4_ + NaOH) and KCl present in the activation solution (Figure S5B). As expected, the amount of carbon (97.2%) increased and the amount of oxygen (2.2%) decreased after the electropolymerization. In addition, a low amount of nitrogen (0.6%) was observed in the spectrum due to the azo group in the structure of CFB (Figure S5C).

**Figure 3:**
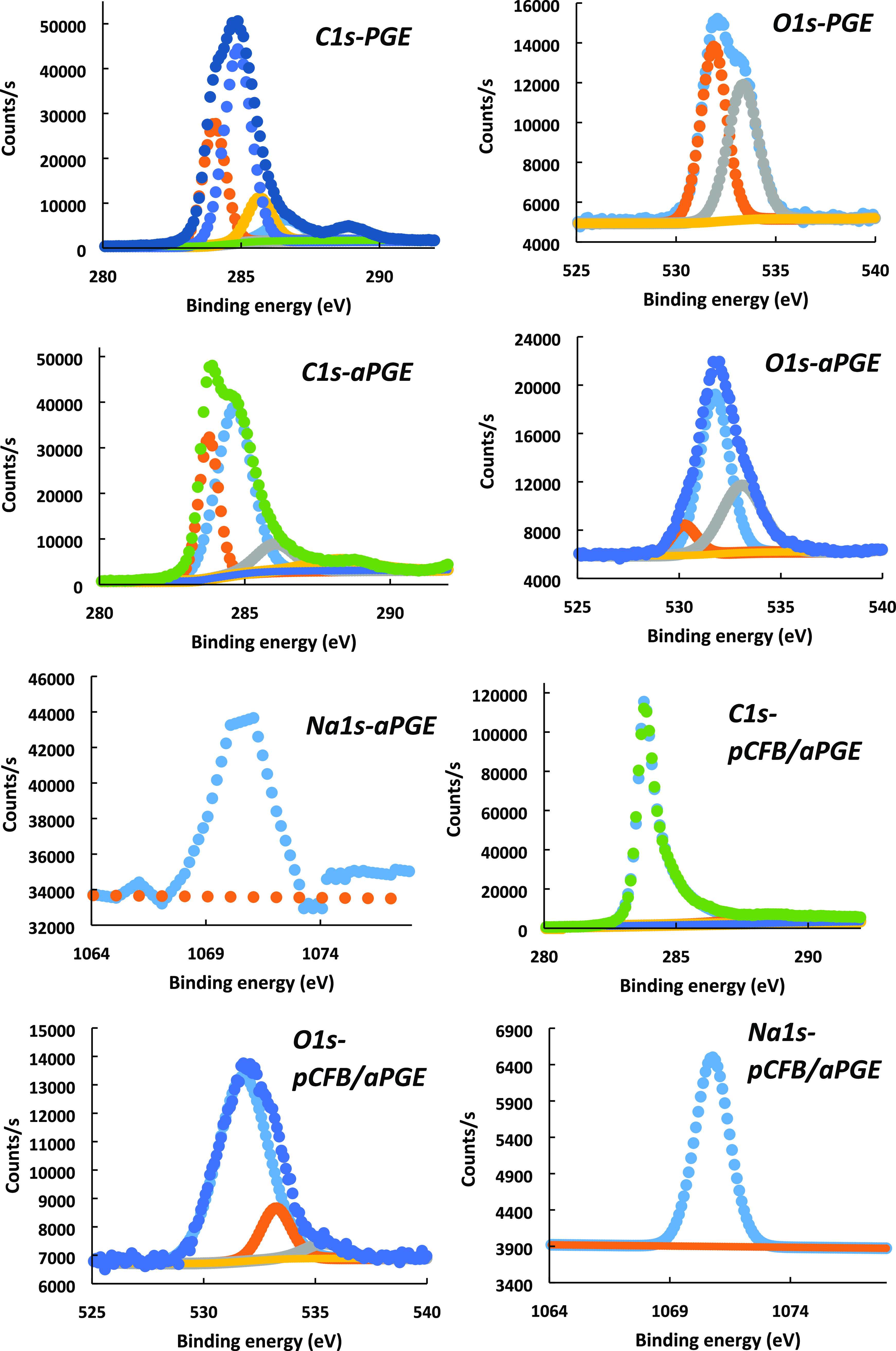

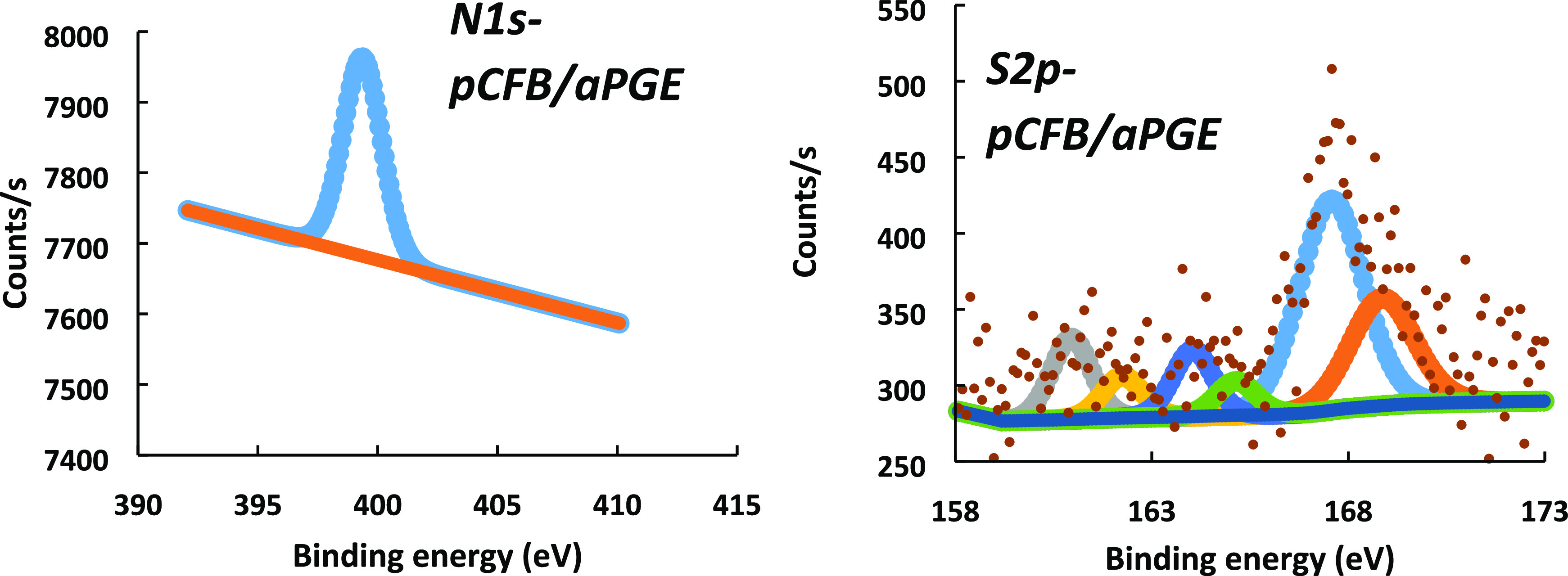
XPS spectra for bare PGE, aPGE, and pCFB/aPGE. XPS analysis: Al Kα gun, 300 μm spot size, 50 eV pass energy, 0.1 eV energy step size.

XPS measurements were fulfilled to acquire more information regarding the characterization of each modification step, and the spectra are given in Figure 3. C 1s bonds between 283.78 and 286.28 eV belong to the C–C and C–O–C structures.^45^ A more than 2-fold increase in the C 1s signal at pCFB/aPGE clearly indicates a carbon-based formation on the surface (i.e., polymer of CFB). O 1s bonds between 531.88 and 532.08 eV correspond to the aliphatic C–O–C structures, and the increase of the O 1s peak at 531.88 eV for aPGE results from the activation procedure of the bare PGE. In addition, the peak that became apparent at 530.48 eV after activation is due to Al_2_O_3_. Although this peak is not observed at the bare PGE with XPS fitting, the characteristic O 1s behavior of Al_2_O_3_ is shown in Figure S6A,B with red circles for both the bare and aPGE, respectively. It is observed that the peak at 530.48 eV disappears with pCFB coating and the characteristic Al_2_O_3_ behavior is lost in the survey spectrum (O 1s region) for pCFB/aPGE (Figure S6C).^46^

The bonds at 533.58 and 533.28 eV are due to the partial aromatic C–O–C structures of graphite,^47^ while the small peak at 535.68 eV for pCFB/aPGE corresponds to the C–O group of CFB during electropolymerization.^48^ Since sodium ion is present in the activation solution and CFB is a sodium salt, Na 1S peaks are observed around 1070.6 eV for aPGE and pCFB/aPGE. One of the two most significant pieces of evidence of polymerization is the appearance of the nitrogen peak at 399.48 eV and the other evidence is the emergence of sulfur peaks between 161.18 and 168.88 eV.^49,50^ S 2p_1/2_ and S 2p_3/2_ bonds at 168.88 and 167.58 eV belong to the SO_3_ group, while the S 2p_1/2_ and S 2p_3/2_ bonds at 165.08 and 162.28 and 164.18 and 161.18 eV correspond to the C-SO_3_ structures. Considering that the sulfur in the hydroxyl-containing naphthalene ring is more partially positive, it could be deduced that the peaks at 165.08 and 164.18 eV belong to the C-SO_3_ group found in this region.^51^

Consequently, largely harmonized CV, EIS, SEM, EDX, and XPS results showed that the pCFB/aPGE was decently and accurately produced for determining the Qn in real samples.

Using the characterization findings and the oxidation reaction of CFB, the possible electropolymerization mechanism and also the oxidation of Qn were proposed in accordance with the literature as depicted in Figure 1A.^52,53^

### 3.2 Cyclic Voltammetric Characteristics of the System

The effective surface areas of aPGE and pCFB/aPGE were calculated from the slope (*I*_p_ – *v*^1/2^) of the Randles–Sevcik equation
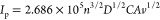
where *I*_p_ is a peak current (A), *n* is the number of electrons transferred, *D* is a diffusion coefficient (cm^2^/s), *C* is a concentration (mol/cm^3^), *A* is an effective surface area (cm^2^), and *v* is a scan rate (V/s). The diffusion coefficient and the number of electrons transferred are 7.6 × 10^–6^ cm^2^/s and 1 for the redox couple ([Fe(CN)_6_]^3–^/[Fe(CN)_6_]^4–^), respectively. The effective surface areas of aPGE and pCFB/aPGE were determined to be 21.3 and 36.9 mm^2^, respectively. It depicted that the electropolymerization greatly increased the effective surface area.

The concentration of the pCFB layer was found from the slope (*I*_p_ – *v*) of the Brown–Anson equation
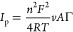
*F* is the Faraday constant (96,485 coulomb/mol), *R* is the gas constant (8.314 J/mol·K), *T* is a temperature (K), and Γ is a surface coverage (mol/cm^2^), which was found as 3.48 nmol/cm^2^.

The type of electrode reaction belonging to the Qn was investigated by performing CV measurements at increasing scan rates between 10 and 1000 mV/s to determine whether the Qn’s relocation to the surface of pCFB/aPGE was diffusion- or adsorption-controlled. The plot of the logarithm of peak height (log(*I*_p_, μA)) and the logarithm of the scan rate (log(*v*, mV/s)) with a slope of 0.81, log(*I*_p_) = 0.810 log(*v*) – 0.024 (*R*^2^: 0.998), showed that the electrode reaction was based on a joint adsorption- and diffusion-controlled process (Figure S7). The adsorption accompanying diffusion was probably due to the Qn’s preference of the polarized pCFB surface.

The typical plot of *E*_p_ (V) versus log(*v*, V/s) was drawn to calculate the apparent charge-transfer coefficient (α) (Figure S8). The slope of *E*_p_ = 0.0511 log(*v*) + 0.2136 (*R*^2^: 0.959) was equal to 2.3*RT*/[(1 – α)*nF*], and α was found as 0.42, thereby depicting how the transition state acted uniformly between the responses of Qn and Qn-*O*-quinone against the applied potential.^20^

The ratio of proton to electron was calculated as 1 from the *E*_p_–pH curve having a slope of −68.6 mV/pH (Figure S9). This coincides with the oxidation of Qn including the 2-electron and 2-proton process reported in the literature (Figure 1A).^9,23^

**Figure 4:**
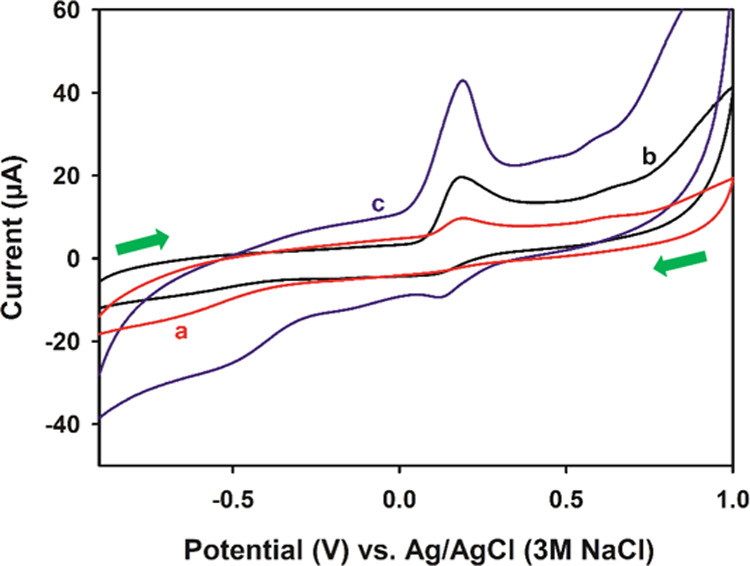
Cyclic voltammograms of (a) bare PGE, (b) aPGE, and (c) pCFB/aPGE in the presence of Qn. Conditions: 150 μM of Qn, 0.03 M (pH 7) of PBS solution, *E*_start_: −1 V, *E*_first_: 1 V, *E*_finish_: −1 V, step amplitude: 3 mV, scan rate: 50 mV/s.

In addition, cyclic voltammograms were recorded with bare PGE, aPGE, and pCFB/aPGE in solution containing Qn to indicate the electrocatalytic effect as shown in Figure 4. The peak currents of Qn oxidation were found as 9.77, 19.92, and 43.02 μA for bare PGE, aPGE, and pCFB/aPGE, respectively. These results demonstrate a peak current increase of about 104% for aPGE over bare PGE, about 116% for pCFB/aPGE over aPGE, and about 340% for pCFB/aPGE over bare PGE. Accordingly, it was found that the pCFB film has quite good electrocatalytic effect on Qn oxidation.

### 3.3 Optimization Studies

The parameters affecting the electropolymerization process including the pencil graphite grade, the concentration of CFB and NaOH, cycle number, and scan rate, and the analysis involving pH, the concentration of the buffer solution, the methanol ratio, and ionic strength were examined with pCFB/aPGE using 0.6 μM of Qn in the respective ranges and specified to be 2H, 1 mM, 0.15 M, 20, and 50 mV/s, and 7, 0.03 M, 20%, and 0, respectively (Figure S10).

### 3.4 Method Validation

**Figure 5:**
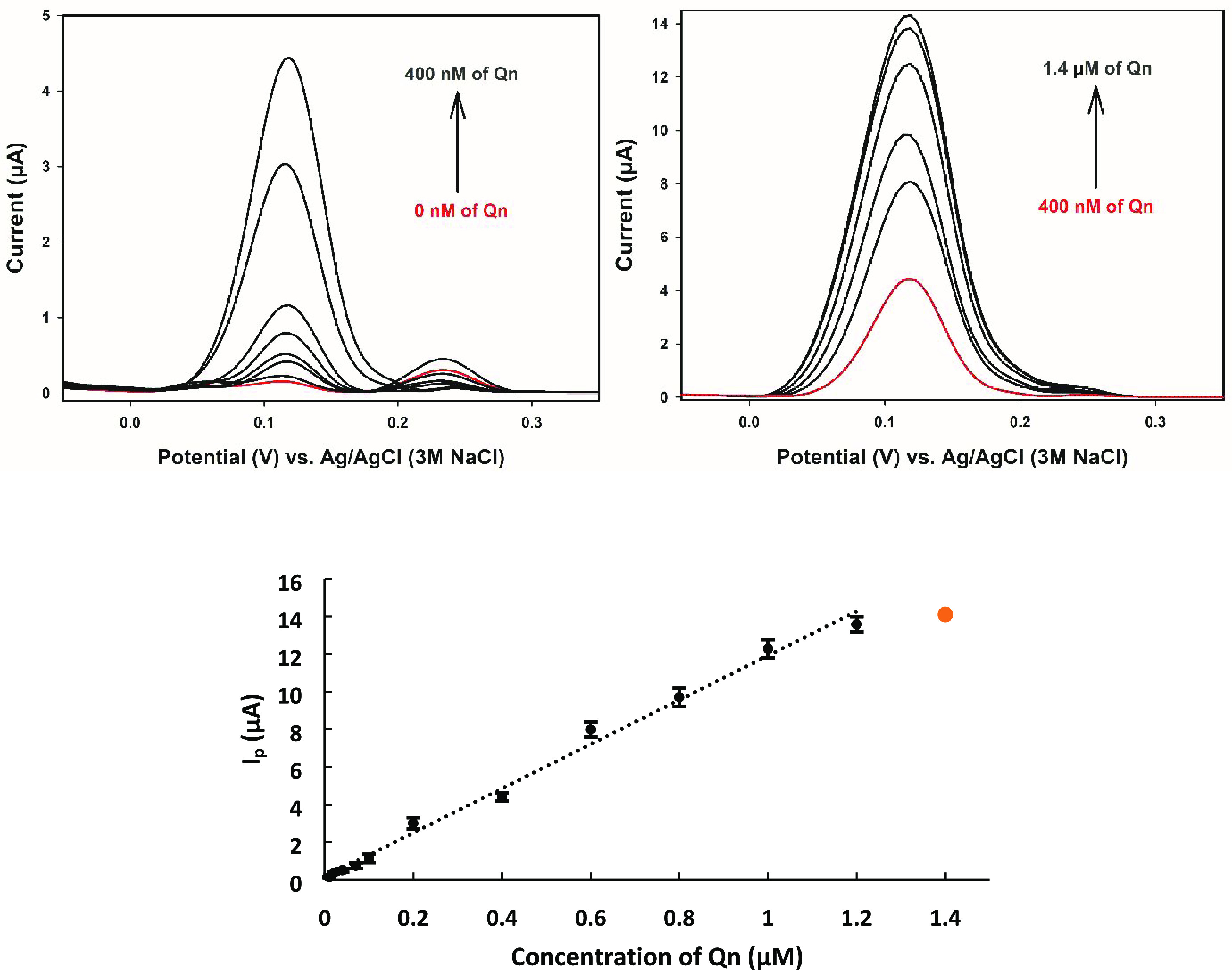
DPV voltammograms and calibration curve belong to the Qn in 0.03 M (pH 7) of PBS solution (*n* = 3 for each concentration). Conditions: *E*_start_: −0.4 V, *E*_finish_: 1.0 V, step amplitude: 5 mV, pulse amplitude: 25 mV, scan rate: 25 mV/s.

The DPV voltammograms and calibration curve belonging to the Qn appear in Figure 5. The peak height of pCFB/aPGE increased proportionally with Qn in 0.03 M (pH 7) PBS solution due to the oxidation of the adjacent hydroxyl groups.^9,23^ The LOD and analytical range for Qn were obtained as 1.9 nM (i.e., from the blank signal, LOD = 3*s*/*m*, where *s* is the standard deviation of the blank solutions (*n* = 6) and *m* is the slope of the calibration curve; the standard error limit is 1.96 for LOD and LOQ calculation) and 0.01–1.2 μM, respectively. The sensitivity of the pCFB/aPGE was calculated as 31.9 μA·μM^–1^·cm^–2^, indicating a better value compared to the literature.^32^

By using six different electrodes at each concentration level, the relative standard deviation (RSD%) values were found as 8, 5, and 3% in the presence of 40 nM, 0.6 μM, and 1.2 μM Qn, respectively. In addition, the repeatability of the pCFB/aPGE was investigated by using the same concentration levels and RSD % values were obtained between 2.2 and 5.4%. The results showed that the produced platform has remarkable reproducibility and repeatability.

Interference studies of various compounds, cations, and anions were carried out in a solution containing 200 nM of Qn using pCFB/aPGE with a criterion of ±5% change in peak height, as detailed in Table S2. Consequently, it was observed that the developed method could tolerate interfering components at least 20-fold more than Qn.

### 3.5 Sample Application

The method was applied to samples of red onion, red cabbage, cranberry, black mulberry, black raisin, and carob using pCFB/aPGE, while the UV–visible spectroscopic method was used as a comparative method. The voltammograms, UV–visible spectra, and calibration curves of real samples, and the results of both voltammetry and UV–visible spectroscopy are shown in Figures S11 and S12 and Table 1, respectively. The recoveries and RSD % values varied from 94.10 to 102.07% and from 0.86 to 3.04%, respectively. The high recovery values in red onion and cranberry are due to their naturally high Qn content. The values calculated by multiplying the results obtained from both methods by the dilution factors were in agreement with the literature (∼250 mg/kg for red onion and ∼130 mg/kg for cranberry).^54,55^

**Table 1:** Sample Application Results (*n* = 6)^a^

	voltammetric measurements			UV–visible spectroscopy measurements				
samples	Qn found (μM)	recovery (%)	relative standard deviation (%)	Qn found (μM)	recovery (%)	relative standard deviation (%)	*F*_experimental_^b^	*t*_experimental_^c^
red onion	204.13 ± 3.08	102.07 ± 1.54	1.51	204.15 ± 2.15	102.07 ± 1.08	1.05	2.05	0.01
cranberry	202.15 ± 3.85	101.07 ± 1.93	1.90	201.95 ± 1.73	100.98 ± 0.86	0.86	4.96	0.11
black mulberry	192.14 ± 6.28	96.07 ± 3.14	3.27	188.21 ± 5.73	94.10 ± 2.86	3.04	1.20	1.13
black raisin	192.02 ± 2.90	96.01 ± 1.45	1.51	193.87 ± 3.75	96.93 ± 1.88	1.94	1.68	0.95
red cabbage	191.83 ± 4.51	95.92 ± 2.26	2.35	191.94 ± 3.78	95.97 ± 1.89	1.97	1.42	0.04
carob	192.26 ± 4.64	96.13 ± 2.32	2.41	193.73 ± 2.59	96.87 ± 1.29	1.34	3.21	0.68

a Conditions: diluted amounts of real samples in 0.03 M (pH 7) of PBS solution, *E*_start_: −0.4 V, *E*_finish_: 1.0 V, step amplitude: 5 mV, pulse amplitude: 25 mV, scan rate: 25 mV/s.

b *F*_critical_ is 5.050 for 5; 5 degrees of freedom.

c *t*_critical_ is 2.228 for 10 degrees of freedom.

The precision and the trueness of the proposed method were evaluated statistically by using UV–visible spectroscopy, and the results showed that the developed method had good accuracy at a 95% confidence interval for all samples because the critical *F* (*F*_critical_) and *t* (*t*_critical_) values exceeded the experimental *F* (*F*_experimental_) and *t* (*t*_experimental_) values (Table 1).

## 4 Conclusions

To the best of our knowledge, a novel, disposable, and cost-effective polymer film-modified electrode, pCFB/aPGE, was designed and produced using CFB as a monomer in the presence of NaOH for the first time and comprehensively characterized by various techniques including CV, EIS, SEM, EDX, and XPS. In addition, a simple, cheap, durable, disposable, and commercially available supporting surface (i.e., PGE) was proposed as an alternative to supporting surfaces such as CPE and GCE, which are more expensive, nondisposable, and require pretreatment steps for Qn determination. The developed platform, pCFB/aPGE, exhibited an extremely electrocatalytic effect toward the oxidation of Qn by enhancing the peak current by about 340% over bare PGE. The pCFB/aPGE achieved an excellent sensitivity (31.9 μA·μM^–1^·cm^–2^, LOD = 1.9 nM) and a wide linear range (0.01–1.2 μM) and had good reproducibility and repeatability between 3–8 and 2.2–5.4% at different concentration levels, respectively. Besides, the electrochemical sensor platform displayed good applicability in detecting Qn in real samples such as red onion, red cabbage, cranberry, black mulberry, black raisin, and carob without being affected by the sample matrix. Voltammetric determination of Qn in these vegetables and fruits other than red onion was made for the first time. The recovery and RSD % values were obtained in the range of 94.10–102.07 and 0.86–3.04%, respectively. The results of the voltammetric method were compared to those of UV–visible spectroscopy, and no statistical difference was found at 95% confidence interval, depicting the excellent accuracy of the proposed method.

## Supporting Information Available

Electrochemical methods for the determination of quercetin, CV, EIS, SEM, EDX, and XPS measurements, optimization and interference studies, and the results of sample application (PDF)

## References

[ref1] Shen P.; Lin W.; Deng X.; Ba X.; Han L.; Chen Z.; Qin K.; Huang Y.; Tu S. Potential Implications of Quercetin in Autoimmune Diseases. Front. Immunol. 2021, 12, 1991 10.3389/fimmu.2021.689044. PMC8260830 34248976

[ref2] Salehi B.; Machin L.; Monzote L.; Sharifi-Rad J.; Ezzat S. M.; Salem M. A.; Merghany R. M.; El Mahdy N. M.; Kılıç C. S.; Sytar O.; Sharifi-Rad M.; Sharopov F.; Martins N.; Martorell M.; Cho W. C. Therapeutic Potential of Quercetin: New Insights and Perspectives for Human Health. ACS Omega 2020, 5, 11849–11872. 10.1021/acsomega.0c01818. 32478277 PMC7254783

[ref3] Shabbir U.; Rubab M.; Daliri E. B.; Chelliah R.; Javed A.; Oh D.-H. Curcumin, Quercetin, Catechins and Metabolic Diseases: The Role of Gut Microbiota. Nutrients 2021, 13, 206 10.3390/nu13010206. 33445760 PMC7828240

[ref4] Carullo G.; Cappello A. R.; Frattaruolo L.; Badolato M.; Armentano B.; Aiello F. Quercetin and Derivatives: Useful Tools in Inflammation and Pain Management. Future Med. Chem. 2017, 9, 79–93. 10.4155/fmc-2016-0186. 27995808

[ref5] Kaul T. N.; Middleton E. Jr.; Ogra P. L. Antiviral Effect of Flavonoids on Human Viruses. J. Med. Virol. 1985, 15, 71–79. 10.1002/jmv.1890150110. 2981979

[ref6] Ansari M. A.; Abdul H. M.; Joshi G.; Opii W. O.; Butterfield D. A. Protective Effect of Quercetin in Primary Neurons against Aβ(1–42): Relevance to Alzheimer’s Disease. J. Nutr. Biochem. 2009, 20, 269–275. 10.1016/j.jnutbio.2008.03.002. 18602817 PMC2737260

[ref7] Lekakis J.; Rallidis L. S.; Andreadou I.; Vamvakou G.; Kazantzoglou G.; Magiatis P.; Skaltsounis A.-L.; Kremastinos D. T. Polyphenols Compounds from Red Grapes Acutely Improve Endothelial Function in Patients with Coronary Heart Disease. Eur. J. Cardiovasc. Prev. Rehabil. 2005, 12, 596–600. 10.1097/01.hjr.0000186622.52863.93. 16319551

[ref8] Tesfaye G.; Hailu T.; Ele E.; Negash N.; Tessema M. Square Wave Voltammetric Determination of Quercetin in Wine and Fruit Juice Samples at Poly (Safranine O) Modified Glassy Carbon Electrode. Sens. Bio-Sens. Res. 2021, 34, 100466 10.1016/j.sbsr.2021.100466.

[ref9] Li J.; Qu J.; Yang R.; Qu L.; de B Harrington P. A Sensitive and Selective Electrochemical Sensor Based on Graphene Quantum Dot/Gold Nanoparticle Nanocomposite Modified Electrode for the Determination of Quercetin in Biological Samples. Electroanalysis 2016, 28, 1322–1330. 10.1002/elan.201500490.

[ref10] Sohrabi M. R.; Darabi G. The Application of Continuous Wavelet Transform and Least Squares Support Vector Machine for the Simultaneous Quantitative Spectrophotometric Determination of Myricetin, Kaempferol and Quercetin as Flavonoids in Pharmaceutical Plants. Spectrochim. Acta, Part A 2016, 152, 443–452. 10.1016/j.saa.2015.07.073. 26241831

[ref11] Yunita E.; Yulianto D.; Fatimah S.; Firanita T. Validation of UV-Vis Spectrophotometric Method of Quercetin in Ethanol Extract of Tamarind Leaf. J. Fundam. Appl. Sci. 2020, 1, 11–18. 10.18196/jfaps.010102.

[ref12] Abdelkawy K. S.; Balyshev M. E.; Elbarbry F. A New Validated HPLC Method for the Determination of Quercetin: Application to Study Pharmacokinetics in Rats. Biomed. Chromatogr. 2017, 31, e3819 10.1002/bmc.3819. 27555122

[ref13] Panchal H.; Amin A.; Shah M. Development of Validated High-performance Thin-layer Chromatography Method for Simultaneous Determination of Quercetin and Kaempferol in Thespesia Populnea. Pharmacognosy Res. 2017, 9, 277 10.4103/0974-8490.210326. 28827970 PMC5541485

[ref14] Liv L.; Yener M.; Karakuş E. A Novel Voltammetric Method for the Sensitive and Selective Determination of Carbonate or Bicarbonate Ions by an Azomethine-H Probe. Anal. Methods 2021, 13, 1925–1929. 10.1039/D1AY00240F. 33913940

[ref15] Liv L.; Nakiboğlu N. Highly Sensitive and Selective Voltammetric Method for the Determination of Hydrazine at a Poly(Eriochrome Black T) Modified Pencil Graphite Electrode (p-EBT/PGE. Anal. Lett. 2022, 55, 688–699. 10.1080/00032719.2021.1960362.

[ref16] Buledi J. A.; Mahar N.; Mallah A.; Solangi A. R.; Palabiyik I. M.; Qambrani N.; Karimi F.; Vasseghian Y.; Karimi-Maleh H. Electrochemical Quantification of Mancozeb through Tungsten Oxide/Reduced Graphene Oxide Nanocomposite: A Potential Method for Environmental Remediation. Food Chem. Toxicol. 2022, 161, 112843 10.1016/j.fct.2022.112843. 35101578

[ref17] Mohanraj J.; Durgalakshmi D.; Rakkesh R. A.; Balakumar S.; Rajendran S.; Karimi-Maleh H. Facile Synthesis of Paper Based Graphene Electrodes for Point of Care Devices: A Double Stranded DNA (DsDNA) Biosensor. J. Colloid Interface Sci. 2020, 566, 463–472. 10.1016/j.jcis.2020.01.089. 32032811

[ref18] Cheraghi S.; Taher M. A.; Karimi-Maleh H.; Karimi F.; Shabani-Nooshabadi M.; Alizadeh M.; Al-Othman A.; Erk N.; Yegya Raman P. K.; Karaman C. Novel Enzymatic Graphene Oxide Based Biosensor for the Detection of Glutathione in Biological Body Fluids. Chemosphere 2022, 287, 132187 10.1016/j.chemosphere.2021.132187. 34509007

[ref19] Liv L.; Nakiboğlu N. Cost-Effective Voltammetric Determination of Boron in Dried Fruits and Nuts Using Modified Electrodes. Food Chem. 2020, 311, 126013 10.1016/j.foodchem.2019.126013. 31855768

[ref20] Olgaç N.; Karakuş E.; Şahin Y.; Liv L. Voltammetric Method for Determining Ferric Ions with Quercetin. Electroanalysis 2021, 33, 2115–2121. 10.1002/elan.202100195.

[ref21] Piovesan J. V.; Spinelli A. Determination of Quercetin in a Pharmaceutical Sample by Square-Wave Voltammetry Using a Poly(Vinylpyrrolidone)-Modified Carbon-Paste Electrode. J. Braz. Chem. Soc. 2014, 25, 517–525. 10.5935/0103-5053.20140019.

[ref22] Manokaran J.; Muruganantham R.; Muthukrishnaraj A.; Balasubramanian N. Platinum- Polydopamine @SiO2 Nanocomposite Modified Electrode for the Electrochemical Determination of Quercetin. Electrochim. Acta 2015, 168, 16–24. 10.1016/j.electacta.2015.04.016.

[ref23] Pereira E. R. de C. V.; Bessegato G. G.; Yamanaka H.; Zanoni M. V. B. Determination of Quercetin by a Siloxane-Polyester/Poly-L-Lysine Nanocomposite Modified Glassy Carbon Electrode. Anal. Lett. 2016, 49, 1398–1411. 10.1080/00032719.2015.1104323.

[ref24] Selvi B.; Sadikoglu M.; Soylu U. I.; Yilmaz S.; Onal A.; Eser F. Sensitive Determination of Quercetin in Onion Peel by Voltammetry using a Poly (4-Aminobenzene Sulfonic Acid) Modified Glassy Carbon Electrode. Anal. Bioanal. Electrochem. 2017, 9, 574–585.

[ref25] Ponnaiah S. K.; Periakaruppan P. A. A Glassy Carbon Electrode Modified with a Copper Tungstate and Polyaniline Nanocomposite for Voltammetric Determination of Quercetin. Microchim. Acta 2018, 185, 524 10.1007/s00604-018-3071-4. 30374580

[ref26] Gutiérrez F.; Ortega G.; Cabrera J. L.; Rubianes M. D.; Rivas G. A. Quantification of Quercetin Using Glassy Carbon Electrodes Modified with Multiwalled Carbon Nanotubes Dispersed in Polyethylenimine and Polyacrylic Acid. Electroanalysis 2010, 22, 2650–2657. 10.1002/elan.201000291.

[ref27] Jin G.-P.; He J.-B.; Rui Z.-B.; Meng F.-S. Electrochemical Behavior and Adsorptive Stripping Voltammetric Determination of Quercetin at Multi-Wall Carbon Nanotubes-Modified Paraffin-Impregnated Graphite Disk Electrode. Electrochim. Acta 2006, 51, 4341–4346. 10.1016/j.electacta.2005.12.011.

[ref28] Erady V.; Mascarenhas R. J.; Satpati A. K.; Detriche S.; Mekhalif Z.; Delhalle J.; Dhason A. A Novel and Sensitive Hexadecyltrimethylammoniumbromide Functionalized Fe Decorated MWCNTs Modified Carbon Paste Electrode for the Selective Determination of Quercetin. Mater. Sci. Eng., C 2017, 76, 114–122. 10.1016/j.msec.2017.03.082. 28482479

[ref29] Salmi Z.; Benmehdi H.; Lamouri A.; Decorse P.; Jouini M.; Yagci Y.; Chehimi M. M. Preparation of MIP Grafts for Quercetin by Tandem Aryl Diazonium Surface Chemistry and Photopolymerization. Microchim. Acta 2013, 180, 1411–1419. 10.1007/s00604-013-0993-8.

[ref30] Sun S.; Zhang M.; Li Y.; He X. A Molecularly Imprinted Polymer with Incorporated Graphene Oxide for Electrochemical Determination of Quercetin. Sensors 2013, 13, 5493–5506. 10.3390/s130505493. 23698263 PMC3690011

[ref31] Arvand M.; Anvari M. A Graphene-Based Electrochemical Sensor for Sensitive Detection of Quercetin in Foods. J. Iran. Chem. Soc. 2013, 10, 841–849. 10.1007/s13738-013-0219-3.

[ref32] Vinothkumar V.; Sangili A.; Chen S.-M.; Veerakumar P.; Lin K.-C. Sr-Doped NiO_3_ Nanorods Synthesized by a Simple Sonochemical Method as Excellent Materials for Voltammetric Determination of Quercetin. New J. Chem. 2020, 44, 2821–2832. 10.1039/C9NJ05660B.

[ref33] Rajendrachari S. Preparation of NiO/ZnO Hybrid Nanoparticles for Electrochemical Sensing of Dopamine and Uric Acid. Chem. Sens. 2012, 2, 1–8.

[ref34] Rajendrachari S. Synthesis of Silver Nanoparticles and Their Applications. Anal. Bioanal. Electrochem. 2013, 5, 455–466.

[ref35] Rajendrachari S. Fabrication of Yttria Dispersed Duplex Stainless Steel Electrode to Determine Dopamine, Ascorbic and Uric Acid Electrochemically by Using Cyclic Voltammetry. Int. J. Sci. Eng. Res. 2016, 7, 1275–1285.

[ref36] Rajendrachari S. Electrochemical Investigation of Duplex Stainless Steel at Carbon Paste Electrode and Its Application to the Detection of Dopamine, Ascorbic and Uric Acid. Int. J. Sci. Eng. Res. 2015, 6, 1863–1871.

[ref37] Rajendrachari S. Effect of Sintering Temperature on the Pitting Corrosion of Ball Milled Duplex Stainless Steel by Using Linear Sweep Voltammetry. Anal. Bioanal. Electrochem. 2018, 10, 349–361.

[ref38] Rajendrachari S.; Kumaraswamy B. E. Biosynthesis of Silver Nanoparticles Using Leaves of Acacia Melanoxylon and Their Application as Dopamine and Hydrogen Peroxide Sensors. Phys. Chem. Res. 2020, 8, 1–18. 10.22036/pcr.2019.205211.1688.

[ref39] Kuskur C. M.; Swamy B. E. K.; Shivakumar K.; Jayadevappa H.; Sharma S. C. Poly (Sunset Yellow) Sensor for Dopamine: A Voltammetric Study. J. Electroanal. Chem. 2019, 840, 52–59. 10.1016/j.jelechem.2019.03.031.

[ref40] Tkach V. V.; Martins J. I. F. P.; Ivanushko Y. G.; Yagodynets P. I. Dye Electropolymerization for Electrochemical Analysis. A Brief Review. Biointerface Res. Appl. Chem. 2022, 12, 4028–4047. 10.33263/BRIAC123.40284047.

[ref41] Šafranko S.; Stanković A.; Asserghine A.; Jakovljević M.; Hajra S.; Nundy S.; Medvidović-Kosanović M.; Jokić S. Electroactivated Disposable Pencil Graphite Electrode – New, Cost-Effective, and Sensitive Electrochemical Detection of Bioflavonoid Hesperidin. Electroanalysis 2021, 33, 1063–1071. 10.1002/elan.202060511.

[ref42] Jin E. Y.; Lim S.; Kim S. oh.; Park Y.-S.; Jang J. K.; Chung M.-S.; Park H.; Shim K.-S.; Choi Y. J. Optimization of Various Extraction Methods for Quercetin from Onion Skin Using Response Surface Methodology. Food Sci. Biotechnol. 2011, 20, 1727–1733. 10.1007/s10068-011-0238-8.

[ref43] Wang S.; Zhang J.; Gharbi O.; Vivier V.; Gao M.; Orazem M. E. Electrochemical Impedance Spectroscopy. Nat. Rev. Methods Primers 2021, 1, 41 10.1038/s43586-021-00039-w.

[ref44] Liv L.; Nakiboğlu N. Voltammetric Determination of Molybdenum Using Polymer Film Modified Pencil Graphite Electrodes. Anal. Lett. 2020, 53, 1155–1175. 10.1080/00032719.2019.1700268.

[ref45] Olgaç N.; Şahin Y.; Liv L. Development and Characterisation of Cysteine-Based Gold Electrodes for the Electrochemical Biosensing of the SARS-CoV-2 Spike Antigen. Analyst 2022, 147, 4462–4472. 10.1039/D2AN01225A. 36052711

[ref46] Strohmeier B. R. Gamma-Alumina (γ-Al2O3) by XPS. Surf. Sci. Spectra 1994, 3, 135–140. 10.1116/1.1247774.

[ref47] Aihara J.-i.; Yamabe T.; Hosoya H. Aromatic Character of Graphite and Carbon Nanotubes. Synth. Met. 1994, 64, 309–313. 10.1016/0379-6779(94)90128-7.

[ref48] Beamson G.; Clark D. T.; Hayes N. W.; Law D. S.-L. Effect of Crystallinity on the XPS Spectrum of Poly(Ethylene Terephthalate). Surf. Sci. Spectra 1994, 3, 357–365. 10.1116/1.1247788.

[ref49] Davies M. High Resolution XPS of Organic Polymers: The Scienta ESCA300 Database (Beamson, G.; Briggs, D.). J. Chem. Educ. 1993, 70, A25 10.1021/ed070pA25.5.

[ref50] Wagner C. D.; Naumkin A. V.; Kraut-Vass A.; Allison J. W.; Powell C. J.; Rumble J. R. Jr. NIST X-ray Photoelectron Spectroscopy Database, version 4.1. http:/srdata.nist.gov/xps/ (accessed October 31, 2022).

[ref51] Siow K. S.; Britcher L.; Kumar S.; Griesser H. J. Sulfonated Surfaces by Sulfur Dioxide Plasma Surface Treatment of Plasma Polymer Films. Plasma Process. Polym. 2009, 6, 583–592. 10.1002/ppap.200950004.

[ref52] Kesavan G.; Chen S.-M. Carbon-Modified Kaolin Clay Using Sugar Dehydration Technique for the Electrochemical Detection of Quercetin. J. Mater. Sci.: Mater. Electron. 2020, 31, 21670–21681. 10.1007/s10854-020-04680-1.

[ref53] Yao H.; Sun Y.; Lin X.; Tang Y.; Huang L. Electrochemical Characterization of Poly(Eriochrome Black T) Modified Glassy Carbon Electrode and Its Application to Simultaneous Determination of Dopamine, Ascorbic Acid and Uric Acid. Electrochim. Acta 2007, 52, 6165–6171. 10.1016/j.electacta.2007.04.013.

[ref54] Häkkinen S. H.; Kärenlampi S. O.; Heinonen I. M.; Mykkänen H. M.; Törrönen A. R. Content of the Flavonols Quercetin, Myricetin, and Kaempferol in 25 Edible Berries. J. Agric. Food Chem. 1999, 47, 2274–2279. 10.1021/jf9811065. 10794622

[ref55] Slimestad R.; Fossen T.; Vågen I. M. Onions: A Source of Unique Dietary Flavonoids. J. Agric. Food Chem. 2007, 55, 10067–10080. 10.1021/jf0712503. 17997520

