# Supplementary material for: Signal-Enhanced Electrochemical Determination of Quercetin with Poly(chromotrope fb)-Modified Pencil Graphite Electrode in Vegetables and Fruits
Source: ACS Omega. 2023 Mar 24;8(13):12522–31. doi: 10.1021/acsomega.3c00599 (PMC10077562; doi:10.1021/acsomega.3c00599)
Supplement: Supplementary file 1 — ao3c00599_si_001.pdf [file ao3c00599_si_001.pdf]

Signal-enhanced Electrochemical Determination of Quercetin with Poly (chromotrope fb) Modified Pencil Graphite Electrode in Vegetables and Fruits

Lokman Liv[a*], Erman Karakuş[b]

[a]Electrochemistry Laboratory, Chemistry Group, The Scientific and Technological Research Council of Turkey, National Metrology Institute, (TUBITAK UME), 41470, Gebze, Kocaeli, Turkey.

[b]Organic Chemistry Laboratory, Chemistry Group, The Scientific and Technological Research Council of Turkey, National Metrology Institute, (TUBITAK UME), 41470, Gebze, Kocaeli, Turkey.

E-mail: lokman.liv@tubitak.gov.tr

Contents **Page**

1. Introduction..2

2. Surface characterization of pCFB/aPGE...3

3. Cyclic voltammetric characteristics of the system..7

4. Optimisation studies..9

4. Method validation..10

5. Sample application...11

1. Introduction

Table S1. Electrochemical methods for determination of quercetin.

Sensing platform	Method	LOD (μM)	Analytical range (μM)	Sample application	Reference
Poly(L-lysine)/polysiloxane-poly(propylene)oxide/glassy carbon electrode	Square wave voltammetry	0.00276	0.01–1	Green tea	Pereira et al., 2016
Poly(vinylpyrrolidone)/carbon paste electrode	Square wave voltammetry	0.17	0.5–5.5	Pharmaceutical formulation	Piovesan & Spinelli, 2014
Poly(sulfanilic acid)/glassy carbon electrode	Differential pulse voltammetry	20.3	70–900	Onion peel	Selvi et al. 2017
Platinum-poly(dopamine)@SiO_2/glassy carbon electrode	Square wave voltammetry	0.016	0.05–0.38	Tea, onion extract, apple juice, human urine and blood plasma	Manokaran et al., 2015
Multi-walled carbon nanotubes/poly(acrylic acid)/glassy carbon electrode	Square wave voltammetry	0.0075	0.1–5	Onion	Gutiérrez et al., 2010
Hexadecyltrimethylammonium bromide/ Fe-multi-walled carbon nanotubes/carbon paste electrode	Differential pulse voltammetry	0.0012	0.06–3000	Wine, coconut water	Erady et al., 2017
Molecularly imprinted polymer–poly(pyrrole)/graphene oxide/glassy carbon electrode	Differential pulse voltammetry	0.048	0.60–15	Apple juice	Sun et al., 2013
Molecularly imprinted polymer–poly(methacrylic acid- ethylene glycol dimethacrylate)/N-methyl diethanol amine/In-Ti oxide electrode	Square wave voltammetry	0.05	0.05–100	Green tea	Salmi et al., 2013
Copper tungstate–polyaniline/glassy carbon electrode	Differential pulse voltammetry	0.0012	0.01–0.50	Urine, blood, green tea, honey, onion, fruit juices	Ponnaiah & Periakaruppan , 2018
Multi-walled carbon nanotubes/choline/paraffin-impregnated graphite disk electrode	Differential pulse voltammetry	0.0048	0.009–40	-	Jin et al., 2006
Graphene/glassy carbon electrode	Differential pulse voltammetry	0.0036	0.006–10, 10–100	Apple, onion	Arvand & Anvari, 2013
Strontium-doped nickel oxide nanorods/glassy carbon electrode	Differential pulse voltammetry	0.00198	0.01–68.5	Apple and grape juice	Vinothkumar et al., 2020
Poly(chromotrope fb)/activated pencil graphite electrode	Differential pulse voltammetry	0.0019	0.01–1.2	Red onion, red cabbage, cranberry, black mulberry, black raisin and carob	This study

2. Surface characterization of pCFB/aPGE

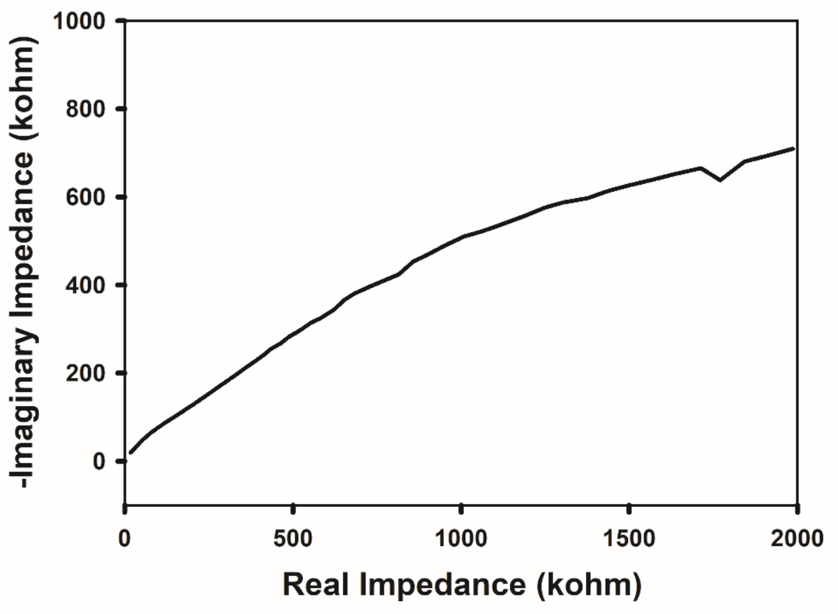

Figure S1. EIS spectra of bare PGE within the frequency range of 0.1-100000 Hz in the presence of 1 mM of $K_3[Fe(CN)_6]$, 1 mM of $K_4[Fe(CN)_6]$ and 0.1 M of KCl.

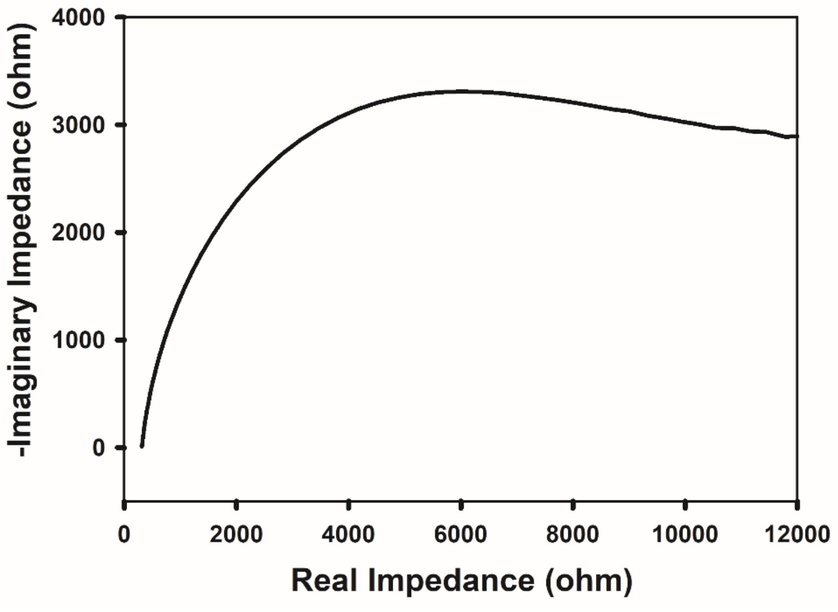

Figure S2. EIS spectra of pCFB/PGE within the frequency range of 0.1-100000 Hz in the presence of 1 mM of $K_3[Fe(CN)_6]$, 1 mM of $K_4[Fe(CN)_6]$ and 0.1 M of KCl.

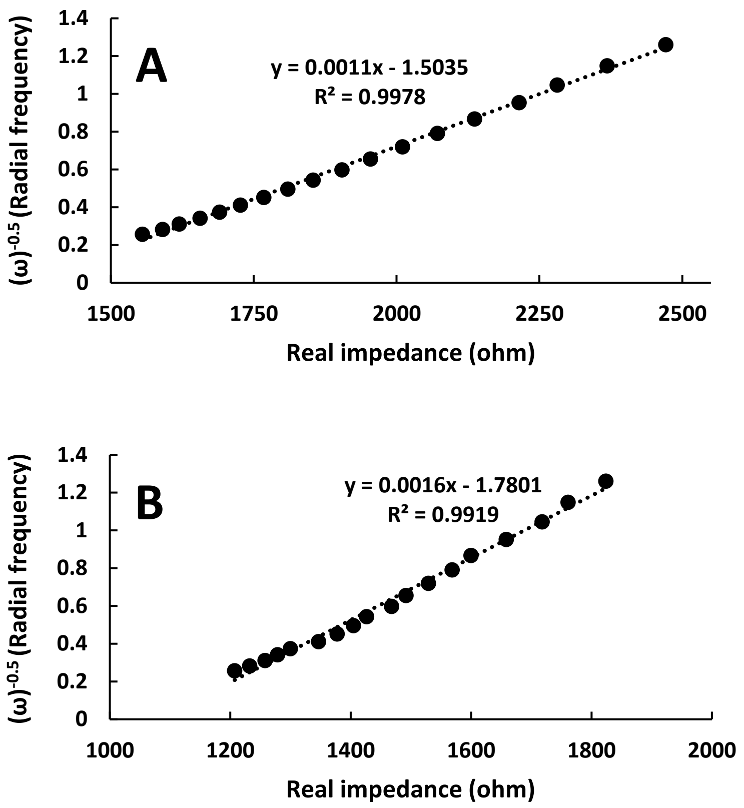

Figure S3. The plots of real impedance and the square root of the inverse of the radial frequency ($\omega^{-0.5}$) for (A) aPGE and (B) pCFB/aPGE.

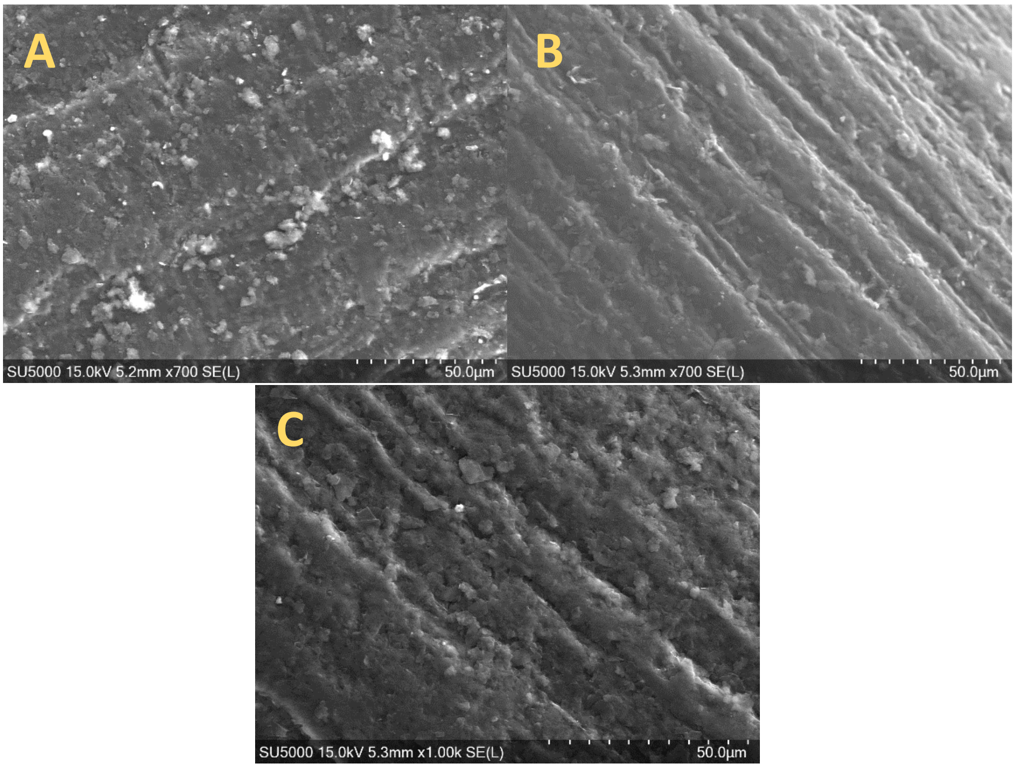

Figure S4. SEM images of (A) bare PGE, (B) aPGE and (C) pCFB/aPGE. (SEM analysis: 15 kV voltage, 30 spot intensity, SE(L) detector).

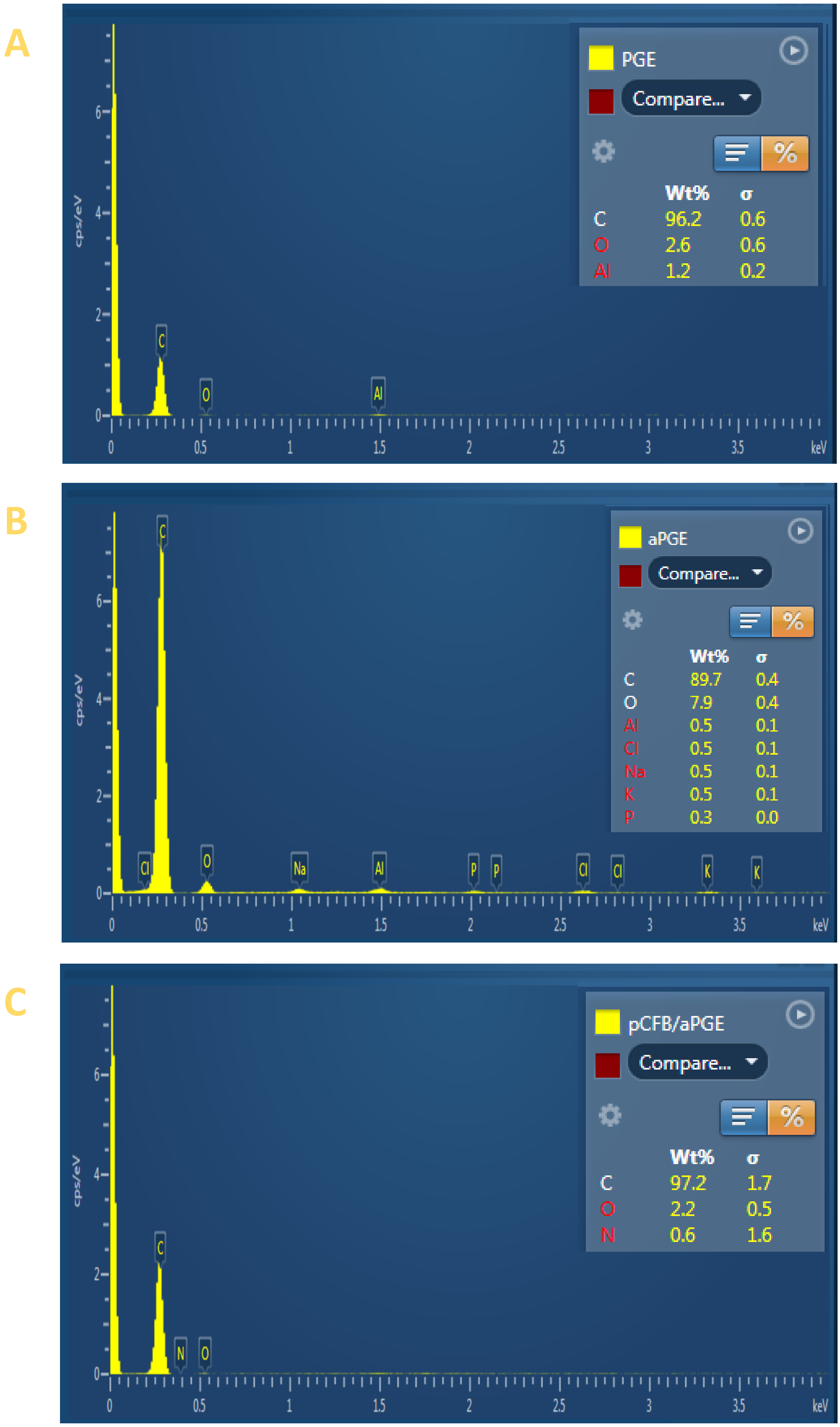

Figure S5. EDX spectra for (A) bare PGE, (B) aPGE and (C) pCFB/aPGE. (EDX analysis: 30 mm^2, AZtec software, mass percentages were given in EDX spectra).

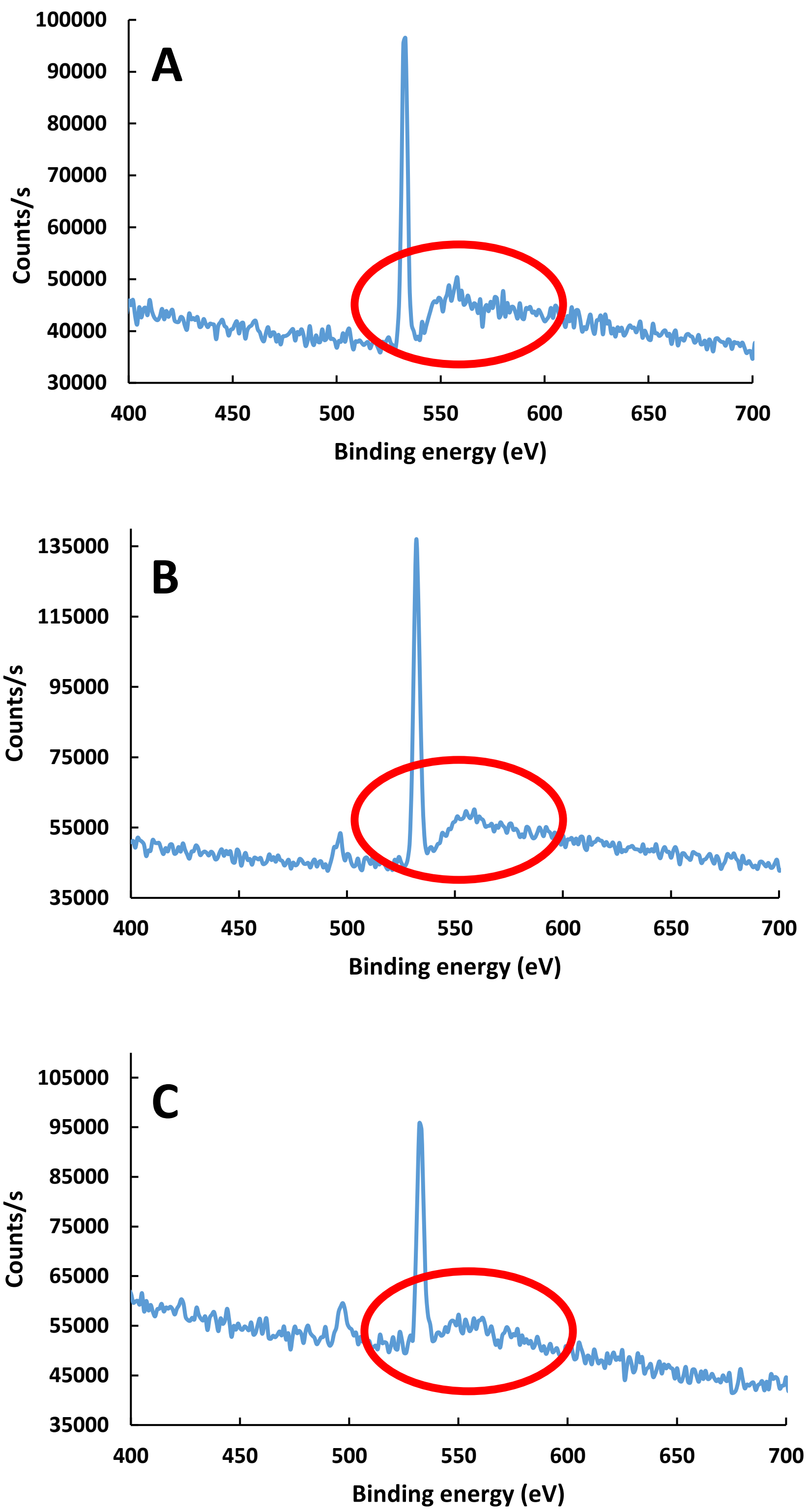

Figure S6. XPS survey spectra for (A) bare PGE–characteristic Al_2O_3 behaviour, (B) aPGE–characteristic Al_2O_3 behaviour and (C) pCFB/aPGE–loss of characteristic Al_2O_3 behaviour. XPS analysis: Al Kα gun, 300 µm spot size, 50 eV pass energy, 0.1 eV energy step size.

3. Cyclic voltammetric characteristics of the system

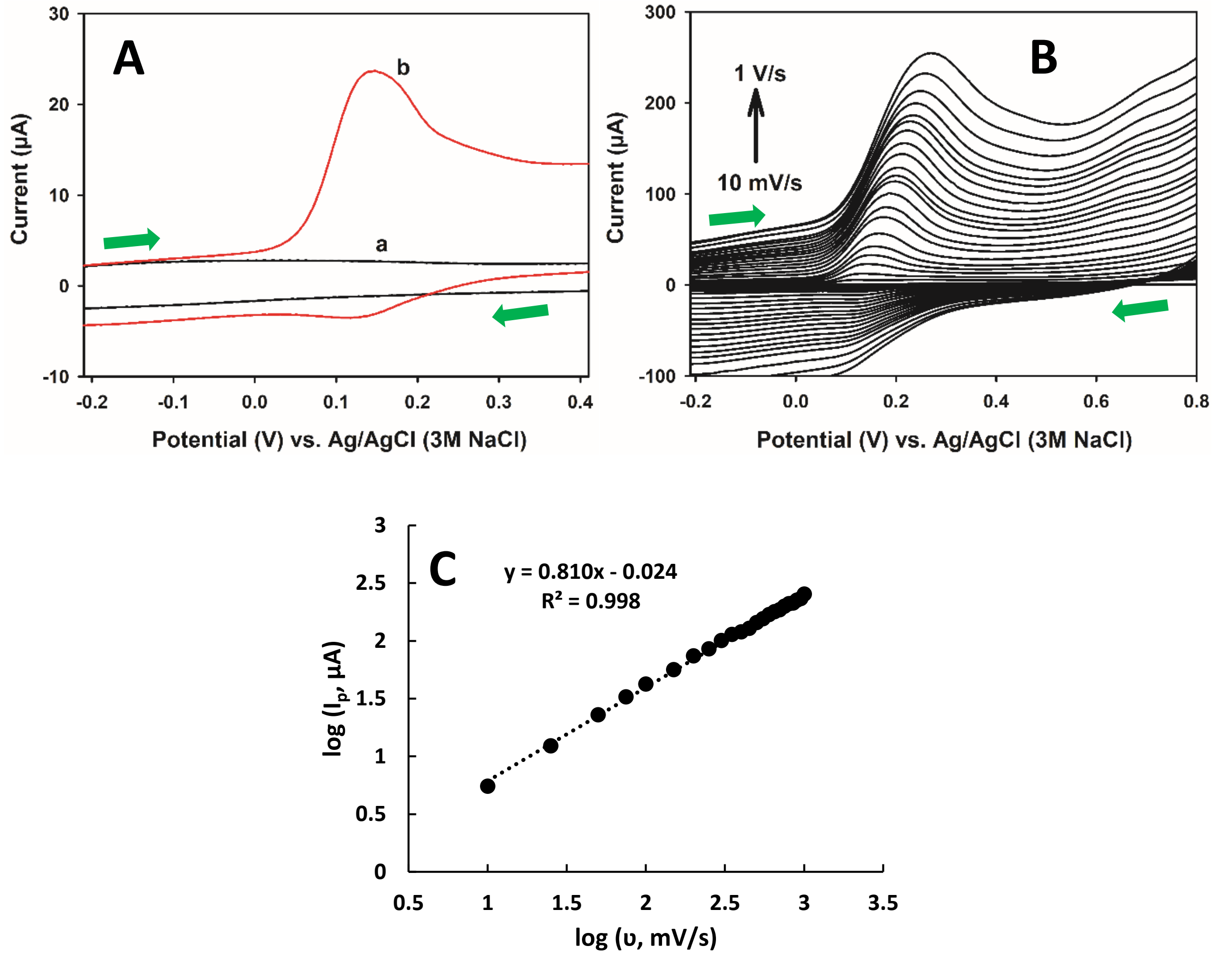

Figure S7. (A) Cyclic voltammograms of pCFB/aPGE at 50 mV/s (a) in the absence and (b) the presence of Qn, (B) cyclic voltammograms of Qn at increasing scan rates using pCFB/aPGE and (C) $\log(I_p) - log(v)$ curve. Conditions: 75 μM of Qn, 0.03 M (pH 7) of PBS solution, E_{start}: -1 V, E_{first}: 1 V, E_{finish}: -1 V, step amplitude: 3 mV.

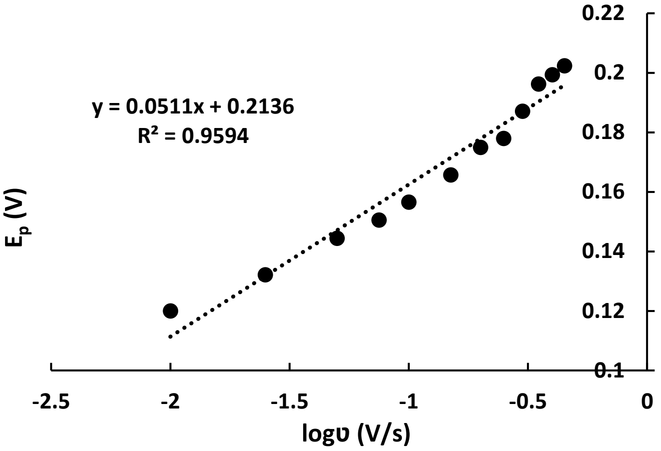

Figure S8. $E_p - log(v)$ curve obtained with pCFB/aPGE. Conditions: 75 µM of Qn, 0.03 M (pH 7) of PBS solution, E_{start}: -1 V, E_{first}: 1 V, E_{finish}: -1 V, step amplitude: 3 mV.

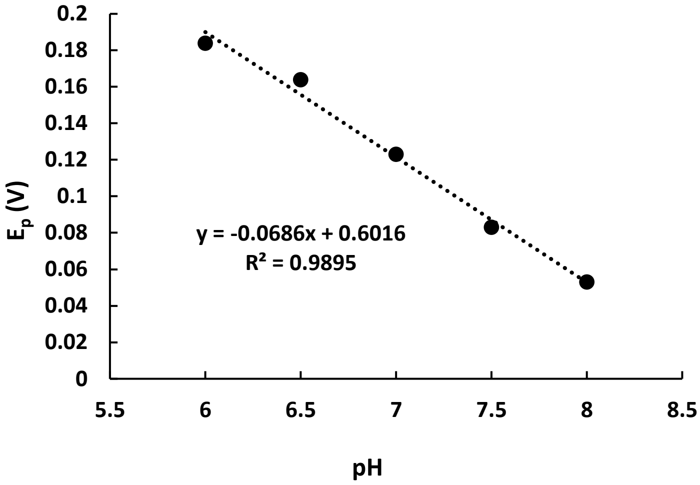

Figure S9. $E_p - pH$ curve obtained with pCFB/aPGE. Conditions: 5 µM of Qn, 0.03 M of PBS solution at different pH values, E_{start}: -0.4 V, E_{finish}: 1.0 V, step amplitude: 5 mV, pulse amplitude: 25 mV, scan rate: 25 mV/s.

4. Optimisation studies

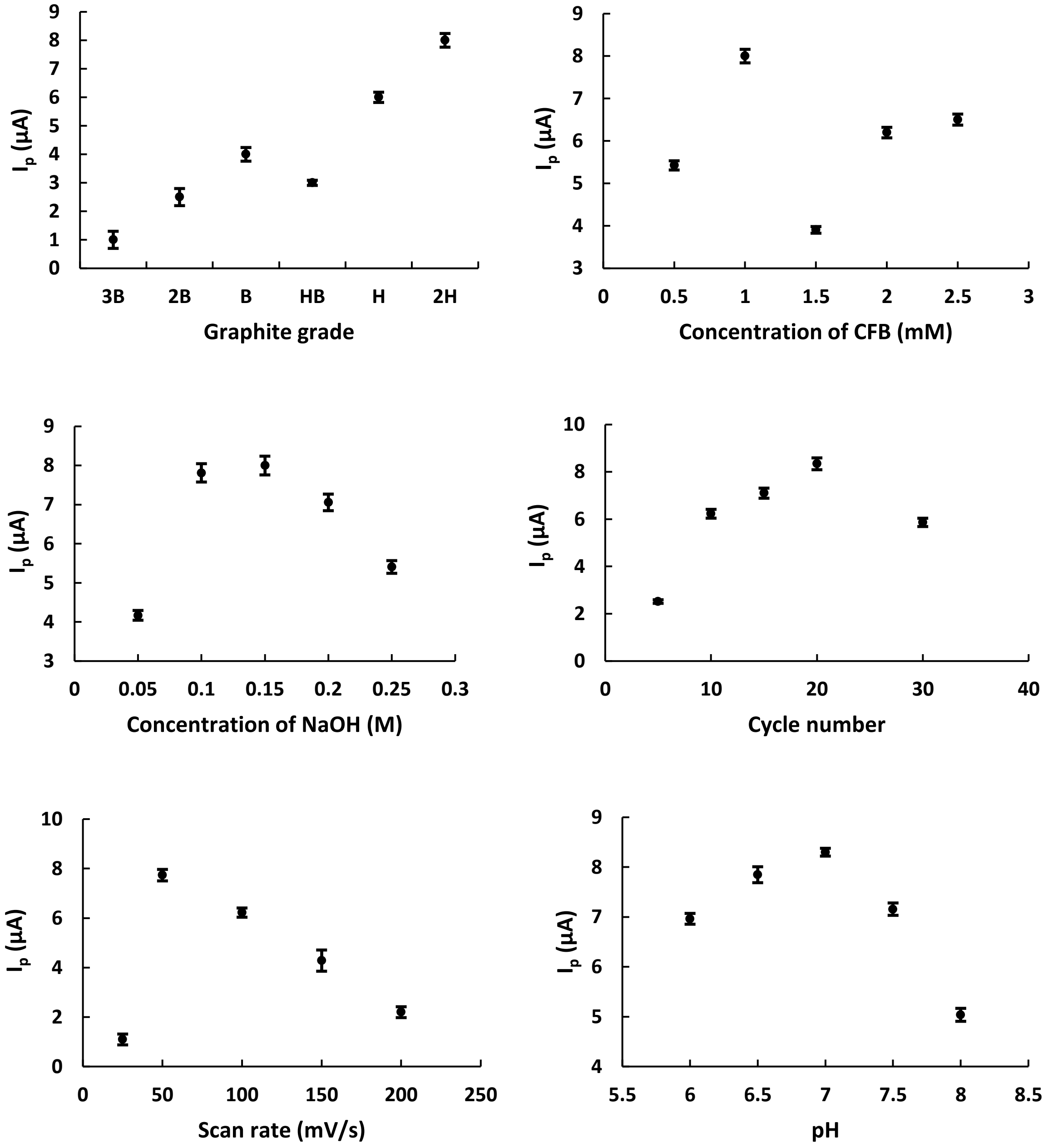

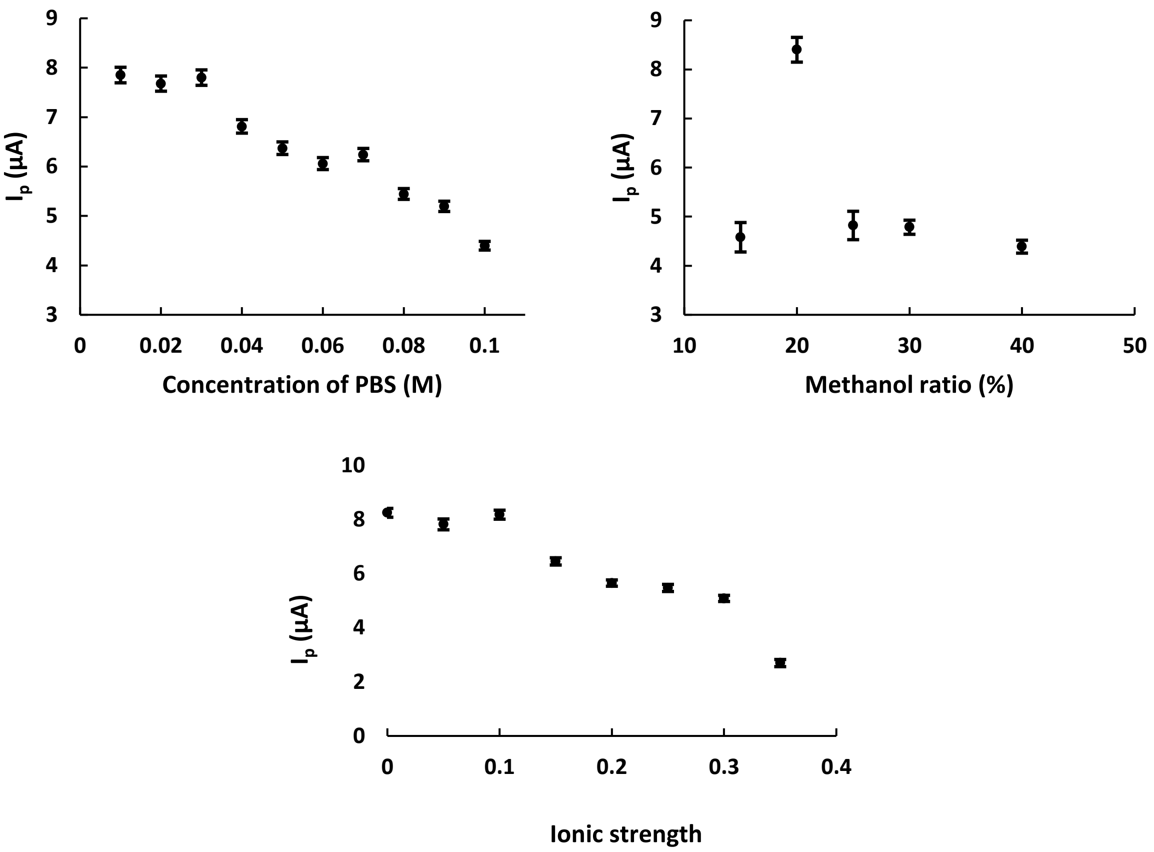

Figure S10. Optimisation studies of the parameters affecting the electropolymerization process including pencil graphite grade, the concentration of CFB and NaOH, cycle number and scan rate, and the analysis involving pH, the concentration of the buffer solution, the methanol ratio and ionic strength. Conditions: 0.6 µM of Qn. E_{start}: -0.4 V, E_{finish}: 1.0 V, step amplitude: 5 mV, pulse amplitude: 25 mV, scan rate: 25 mV/s.

5. Method validation

Table S2. Interference studies and tolerable ratios of various compounds, cations and anions for voltammetric determination of Qn with pCFB/aPGE. Conditions: 0.03 M of pH 7 PBS, 20% methanol, 200 nM of Qn. E_{start}: -0.4 V, E_{finish}: 1.0 V, step amplitude: 5 mV, pulse amplitude: 25 mV, scan rate: 25 mV/s.

Interferences	Tolerable ratio[a]
NO_2^-, ClO_4^-, $S_2O_3^{2-}$, Na^+, K^+, Cl^-	2000
NO_3^-, SO_4^{2-}, glucose, sucrose	1000
Ca^{2+}, Mg^{2+}, Al^{3+}, Fe^{3+}	500
Ascorbic acid, glycine, L-cysteine	250
Citric acid	30
Dopamine	20

[a] The concentration of each interference was obtained by multiplying the tolerable ratio with the Qn concentration value of 200 nM.

6. Sample application

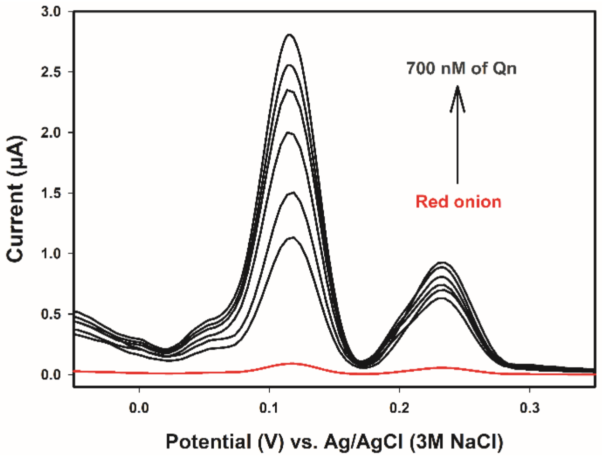

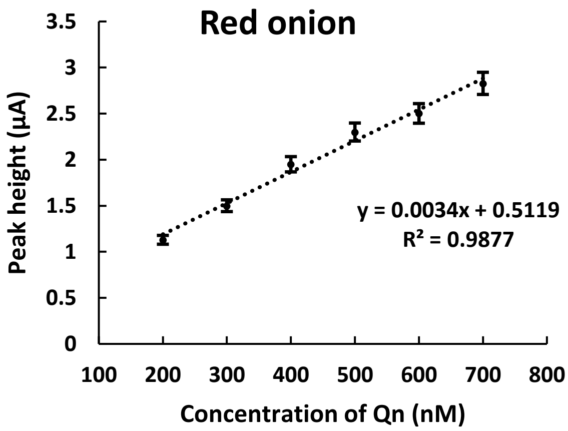

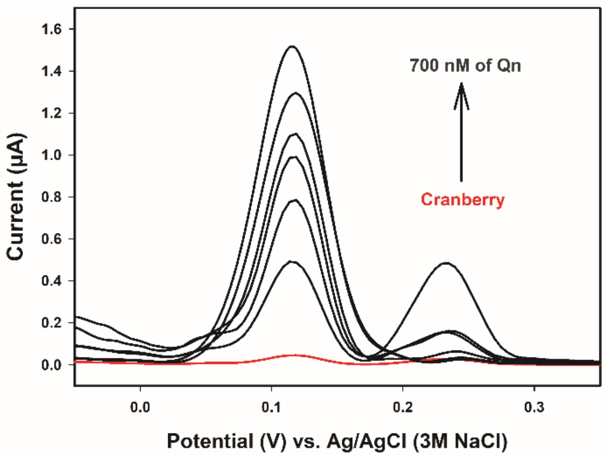

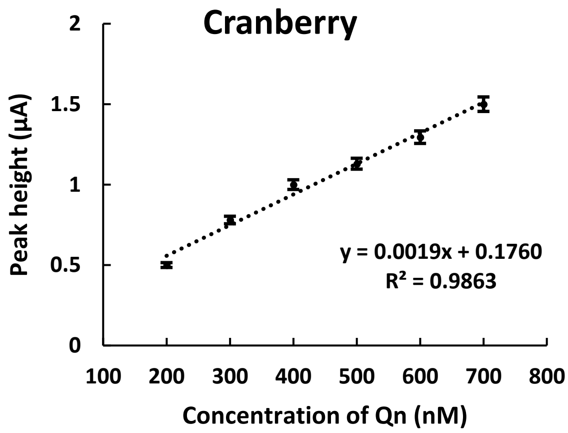

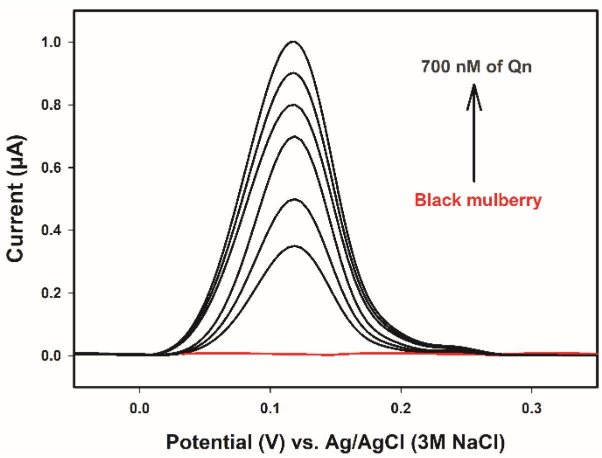

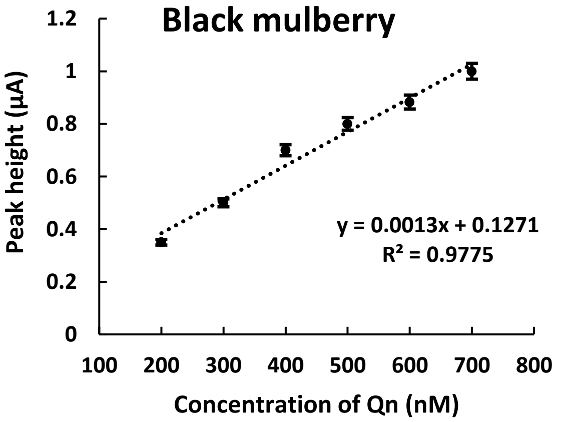

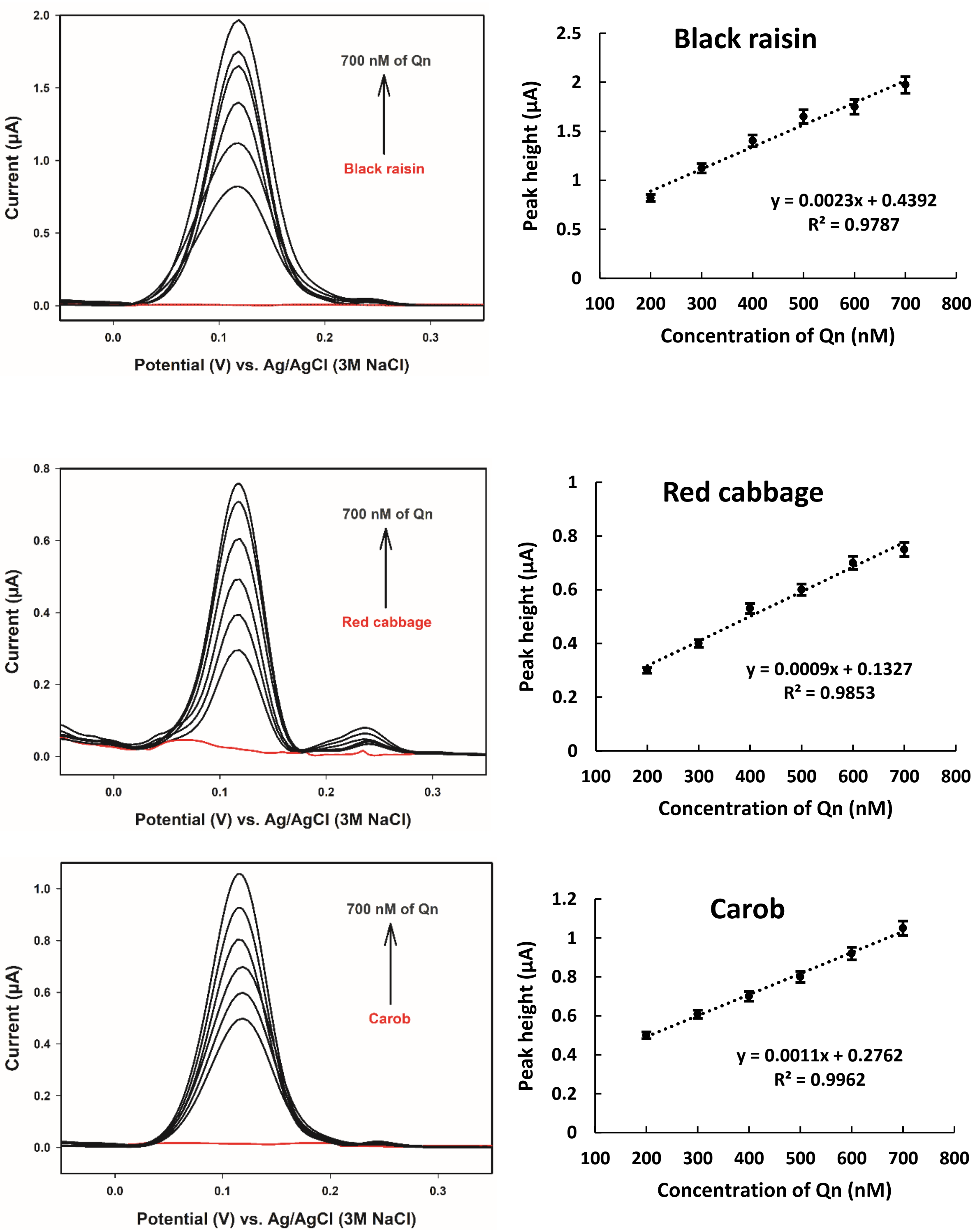

Figure S11. DPV voltammograms and calibration curves belong to the samples of red onion, red cabbage, cranberry, black mulberry, black raisin and carob. Conditions: 500-fold diluted amounts of real samples in 0.03 M (pH 7) of PBS solution (n= 3 for each concentration). E_{start}: -0.4 V, E_{finish}: 1.0 V, step amplitude: 5 mV, pulse amplitude: 25 mV, scan rate: 25 mV/s.

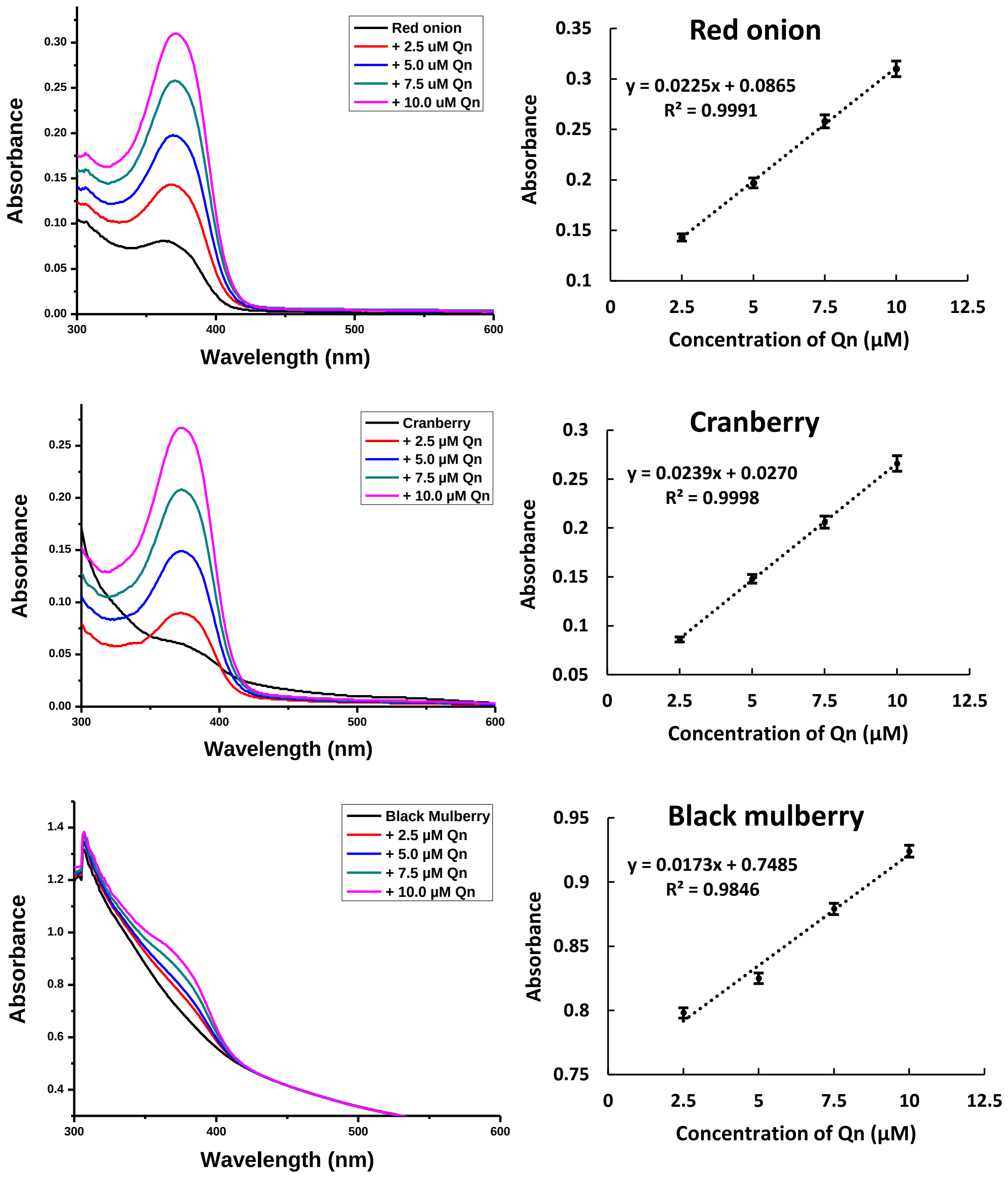

Red onion
+ 2.5 uM Qn
+ 5.0 uM Qn
+ 7.5 uM Qn
+ 10.0 uM Qn
Absorbance
Wavelength (nm)
Red onion
y = 0.0225x + 0.0865
R² = 0.9991
Absorbance
Concentration of Qn (μM)
Cranberry
+ 2.5 μM Qn
+ 5.0 μM Qn
+ 7.5 μM Qn
+ 10.0 μM Qn
Absorbance
Wavelength (nm)
Cranberry
y = 0.0239x + 0.0270
R² = 0.9998
Absorbance
Concentration of Qn (μM)
Black Mulberry
+ 2.5 μM Qn
+ 5.0 μM Qn
+ 7.5 μM Qn
+ 10.0 μM Qn
Absorbance
Wavelength (nm)
Black mulberry
y = 0.0173x + 0.7485
R² = 0.9846
Absorbance
Concentration of Qn (μM)

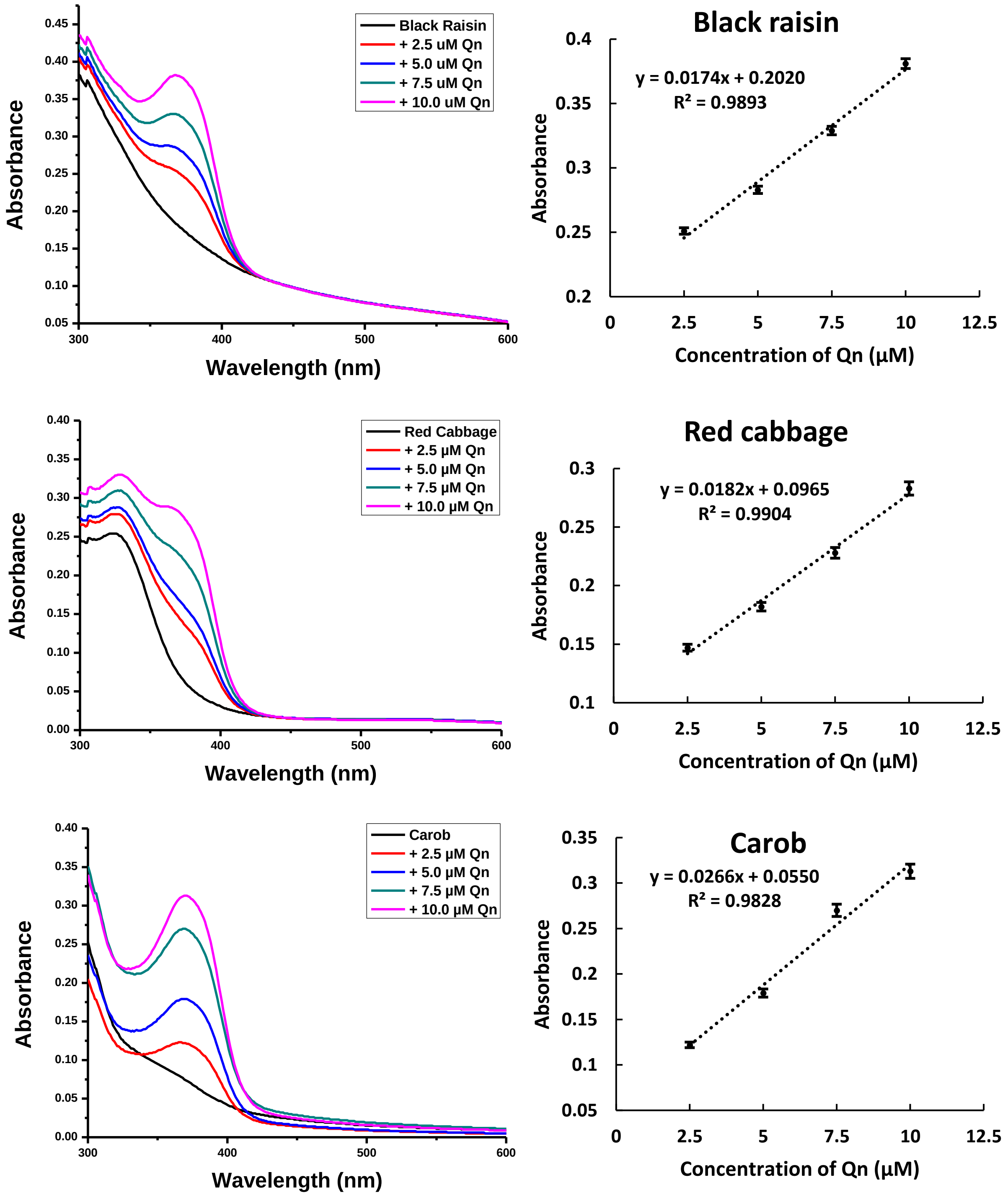

Figure S12. UV-visible spectra and calibration curves belong to the samples of red onion, red cabbage, cranberry, black mulberry, black raisin and carob. Conditions: 40-fold diluted amounts of real samples in 1:1 (v/v) ethanol:water (n= 3 for each concentration), path length: 10.0 mm, sample volume: 700 µL, absorbance measurement range: 300 nm–700 nm.
